# Optimizing androgen receptor prioritization using high-throughput assay-based activity models

**DOI:** 10.3389/ftox.2024.1347364

**Published:** 2024-03-11

**Authors:** Ronnie Joe Bever, Stephen W. Edwards, Todor Antonijevic, Mark D. Nelms, Caroline Ring, Danni Harris, Scott G. Lynn, David Williams, Grace Chappell, Rebecca Boyles, Susan Borghoff, Kristan J. Markey

**Affiliations:** ^1^ U.S. Environmental Protection Agency, Washington, DC, United States; ^2^ RTI International, Research Triangle Park, NC, United States; ^3^ ToxStrategies, Katy, TX, United States; ^4^ ToxStrategies, Austin, TX, United States; ^5^ ToxStrategies, Asheville, NC, United States; ^6^ ToxStrategies, Research Triangle Park, NC, United States

**Keywords:** endocrine disruption, androgen receptor, computational toxicology, highthroughput screening, tiered testing

## Abstract

**Introduction:** Computational models using data from high-throughput screening assays have promise for prioritizing and screening chemicals for testing under the U.S. Environmental Protection Agency’s Endocrine Disruptor Screening Program (EDSP). The purpose of this work was to demonstrate a data processing method for the determination of optimal minimal assay batteries from a larger comprehensive model, to provide a uniform method of evaluating the performance of future minimal assay batteries compared with the androgen receptor (AR) pathway model, and to incorporate chemical cluster analysis into this evaluation. Although several of the assays in the AR pathway model are no longer available through the original vendor, this approach could be used for future evaluations of minimal assay models for prioritization and screening.

**Methods:** We compared two previously published models and found that an expanded 14-assay model had higher sensitivity for antagonists, whereas the original 11-assay model had slightly higher sensitivity for agonists. We then investigated subsets of assays in the original AR pathway model to optimize overall testing strategies that minimize cost while maintaining sensitivity across a broad chemical space.

**Results and Discussion:** Evaluation of the critical assays across subset models derived from the 14-assay model identified three critical assays for predicting antagonism and two critical assays for predicting agonism. A minimum of nine assays is required for predicting agonism and antagonism with high sensitivity (95%). However, testing workflows guided by chemical structure–based clusters can reduce the average number of assays needed per chemical by basing the assays selected for testing on the likelihood of a chemical being an AR agonist, according to its structure. Our results show that a multi-stage testing workflow can provide 95% sensitivity while requiring only 48% of the resources required for running all assays from the original full models. The resources can be reduced further by incorporating *in silico* activity predictions.

**Conclusion:** This work illustrates a data-driven approach that incorporates chemical clustering and simultaneous consideration of antagonism and agonism mechanisms to more efficiently screen chemicals. This case study provides a proof of concept for prioritization and screening strategies that can be utilized in future analyses to minimize the overall number of assays needed for predicting AR activity, which will maximize the number of chemicals that can be tested and allow data-driven prioritization of chemicals for further screening under the EDSP.

## 1 Introduction

Perturbation of hormonal balance can result in adverse effects in development and reproduction, increase cancer risk, and affect the immune and nervous systems (Casals-Casas and Desvergne, 2011). The U.S. Environmental Protection Agency’s (EPA’s) Endocrine Disruptor Screening Program (EDSP) was created to prioritize, screen, and test chemicals that potentially interfere with estrogen, androgen, or thyroid hormone–related pathways using a two-tiered battery of *in vitro* and *in vivo* assays and tests (U.S. EPA, 1998b; U.S. EPA, 1998a). Because screening using the Tier 1 battery can be expensive and time consuming, the EDSP developed EDSP for the 21st Century (EDSP21), which relies on computational toxicology and high-throughput screens (U.S. EPA, 2011; U.S. EPA, 2015; U.S. EPA, 2023).

EPA developed a computational network model to detect androgen receptor (AR) agonism and antagonism in the ToxCast/Tox21 subset of chemicals. Initially, an AR pathway model with 11 high-throughput screening ToxCast and Tox21 *in vitro* assays (11-assay Kleinstreuer model) was developed to estimate chemicals’ agonist and antagonist AR activity (Kleinstreuer et al., 2017). The largest area under the curve (AUC) value identifies the chemical modes (antagonist, agonist, or interference). For example, if the model predicts, for a particular chemical, that the agonist branch AUC value is above 0.1 and it is higher than the antagonist branch AUC value, then the model essentially predicts that a chemical produces an agonist effect. The 11-assay model was later revised and expanded by three additional assays (Judson et al., 2020).

Based on the results, the AR model described by Kleinstreuer et al. (2017) has been proposed as an alternative for the current low-throughput androgen screening assays in the EDSP Tier 1 battery (U.S. EPA, 2017; U.S. EPA, 2014; U.S. EPA, 2015; U.S. EPA, 2023). The EDSP Universe of Chemicals (UoC) comprises approximately 10,000 substances that potentially need to be screened for endocrine bioactivity (U.S. EPA, 2012). All combinations of 2–14 assays (a total of 2^14^–15 = 16,369 combinations) were analyzed by Judson et al. (2020) and calculated pathway AUC values for agonist, antagonist and other modes for all chemicals. So, subsequent analyses identified subset models with as few as five to six assays that deliver comparable AR activity predictions to the expanded 14-assay AR model (Judson et al., 2020). This allows for more cost-effective prioritization of chemicals for Tier 1 screening.

The AR models were previously evaluated using balanced accuracy, which is generally a good choice for evaluating binary classifiers. However, this affords equal weight to sensitivity and specificity, whereas sensitivity is more important for prioritization and screening to avoid false negatives. In particular, most of the AR subset models have higher specificity scores than sensitivity scores for the detection of agonism and antagonism effects. For example, Supplementary Tables S3, S4 from Judson et al. (2020) show that only 18 subset models in agonist mode and 125 subset models in antagonist mode have higher sensitivity than specificity.

The current work is focused on identifying assay batteries that include agonist and antagonist subset models that could be used for prioritizing chemicals from the EDSP UoC for EDSP screening based upon potential AR effects. We adjusted the evaluation criteria (specificity >85% and sensitivity >95% across all chemicals in both AR modes) to minimize the number of false negatives produced in this initial round of testing. We first compared the original 11-assay AR model from (Kleinstreuer et al., 2017) with the expanded 14-assay model from Judson et al. (2020), then evaluated the importance of individual assays across the AR subset models, and identified subset models that met our specified criteria. We also investigated the performance of subset models without NovaScreen (NVS) receptor-binding assays, which are no longer available. Finally, we evaluate strategies that leverage chemical structure and activity predictions from subset models to prioritize chemicals from the EDSP UoC for screening.

This exercise illustrates the use of data processing to help choose critical assays from a comprehensive model for optimal screening batteries. Some of the assays investigated in this case study (the NVS receptor-binding assays) are no longer available. However, assays that measure receptor binding with performance (specificity, sensitivity, variability) similar to that of the NVS assays may be substituted and be expected to yield similar battery results. Furthermore, chemical cluster analysis was used in this evaluation and is an important consideration in future analyses for coverage of the EDSP UoC and possible refinements to the regulatory screening process.

## 2 Methods

The code for all analyses described below can be found at: https://github.com/USEPA/edsp-ar-subset-model-analysis.

### 2.1 Data sources

#### 2.1.1 EDSP UoC

The EDSP UoC is a list of approximately 10,200 substances, as defined under the Federal Food, Drug, and Cosmetic Act and the Safe Drinking Water Act 1996 amendments. To facilitate the analysis, the EPA authors provided a computable version of the published EDSP UoC (U.S. EPA, 2012).^1^

#### 2.1.2 ToxCast/Tox21 high-throughput screening data

The high-throughput screening data contained within ToxCast/Tox21 is a set of approximately 9,500 substances that have been tested in up to approximately 1,400 assays as part of the ToxCast/Tox21 program. Information on whether a chemical-assay pair was tested as part of the ToxCast/Tox21 program was downloaded as a comma-separated values file associated with EPA’s invitrodb v3.2 (U.S. EPA, 2020). Meanwhile, assay data including the chemical-assay AC50 values, binary hit call (i.e., the binary representation of activity [1] or inactivity [0] for a given chemical-assay pair), and chemical-specific cytotoxicity point were extracted from invitrodb v3.3 using version 2.0 of the tcpl R package (Filer et al., 2016).

#### 2.1.3 AR pathway model data as AUC scores

A total of 1,820 chemicals tested in all 14 AR-related high-throughput *in vitro* assays present in ToxCast/Tox21 were used to develop the full 14-assay AR model and the associated subset AR models present in Judson et al. (2020) (herein called the AR model set). These 1,820 chemicals are a subset of the 1,855 chemicals used to create the original 11-assay AR model developed by Kleinstreuer et al. (2017).

The R package “ARminassaymodel” (Judson et al., 2020) was used to generate the chemicals’ AR activity predictions. The package accompanies the Judson et al. (2020) paper, which defines the updated 14-assay AR model and all subset models. The “ARminassaymodel” package was downloaded from EPA’s FTP site [ftp://anonymous@newftp.epa.gov/COMPTOX/STAFF/rjudson/publications/Judson%20AR%202019/ARmodelForPublication.zip, accessed September 2020; these data now are available at (Rpubs, 2023)]. After download, the package was set to query the ToxCast invitrodb v3.3 (U.S. EPA, 2020).

### 2.2 Chemicals and AR subset models

#### 2.2.1 Chemical clustering data

As described in our companion article, a study was performed that investigated the nature and structural classes of 10,272 substances in the EDSP UoC and their alignment with EDSP21 (Nelms et al., 2023). Briefly, structural information, in the form of simplified molecular-input line-entry system (SMILES) strings and quantitative structure activity relationship (QSAR)-ready SMILES strings, for the EDSP UoC and AR model substances (Judson et al., 2017; Mansouri et al., 2020) was downloaded from EPA’s CompTox Chemicals Dashboard (Williams et al., 2017). Separate ToxPrint fingerprint representations (Yang et al., 2015) were created using the SMILES and QSAR-ready SMILES strings for each substance in the EDSP UoC and AR model set. The EDSP UoC substances were clustered by calculating the Tanimoto distance (D) between every pair of chemicals using the ToxPrint fingerprints created from the SMILES strings. The Tanimoto coefficient ranges from 0 (no match between fingerprints) to 1 (full match between fingerprints). Next, Ward’s hierarchical clustering algorithm was used to create a hierarchical cluster tree for the entire EDSP UoC. This hierarchical tree was cut at the height of 1 to generate the final chemical clusters. Once the clustering assignment of the EDSP UoC was completed, chemicals in the AR model set were assigned one of the EDSP clusters according to the k-nearest neighbors classification algorithm, where *k* = 1.

#### 2.2.2 Prediction of chemical activities

We investigated the 1,820 chemicals from Judson et al. (2020) that had been tested in all 14 AR-related assays as part of the ToxCast/Tox21 program. For these chemicals, a total of 16,368 subset models and the 14-assay AR model were evaluated. A subset model calculated an AUC metric for each chemical in agonist, antagonist, and interference pathways based upon the assays present in the model. AUC predictions in the full agonist pathway (R1) are associated with chemical activities in assays A1, A2, A3, A4, A5, A6, A7, A8, A9, A10, and A11 [for assay endpoint name definitions, see Table 1 in (Judson et al., 2020)]. AUC predictions in the full antagonist pathway (R2) are associated with chemical activities in assays A1, A2, A3, A4, A5, A6, A12, A13, and A14. However, these pathways are truncated in subset models depending on a particular combination of assays in the subset model. For example, the subset model A10000000001111 contains assays A1, A11, A12, A13, and A14. Therefore, its agonist pathway is composed of only assays A1 and A11. Similarly, its antagonist pathway contains only assays A1, A12, A13, and A14. This article follows the same subset model nomenclature as in Judson et al. (2020).

**TABLE 1:** Comparison between the Kleinstreuer 11-assay AR model and the 14-assay AR model.

Comparison	Number of estimated chemicals	
	Antagonist	Agonist
Both models estimate chemical activity	173	21
Both models estimate chemical inactivity	1,560	1,777
14-assay model estimates activity and the 11-assay model estimates inactivity	74	7
14-assay model estimates inactivity and the 11-assay model estimates activity	10	12

#### 2.2.3 Binarization of agonist and antagonist AUC values

Binarization of agonist and antagonist AUC values uses the same schema as Judson et al. (2020). AUC values less than the cutoff of 0.1 were set to 0 to denote inactive calls. AUC values at or above 0.1 were set to 1 to indicate active calls. According to Judson et al. (2020), the cutoff of 0.1 matches the upper testing limit of ∼200 µM within the *in vitro* assays.

### 2.3 Minimizing the number of assays for testing

#### 2.3.1 Evaluation of subset models by comparing them with the 14-assay model

Based on binarized AUC values, the results of the subset models were compared with the 14-assay model using sensitivity and specificity metrics. In our setup, sensitivity describes how well a subset model detects true positives (TP) compared with all positives identified by the 14-assay model [i.e., TP + false negatives (FN)]. Specificity describes how well a subset model detects true negatives (TN) compared with all negatives determined by the 14-assay model [i.e., TN + false positives (FP)] (Florkowski, 2008; Trevethan, 2017). Sensitivity and specificity were expressed using their mathematical definitions: sensitivity = TP/(TP + FN) and specificity = TN/(TN + FP). They were calculated for each subset model across all chemicals.

#### 2.3.2 Identification of subset models (assays) suitable for AR prioritization

The approach optimizes the number of assays in the testing battery by taking a union of the assays required for the best agonist and antagonist models. The full set of assays used at each testing step will be referred to as the assay battery, whereas the set of assays used for modeling agonist or antagonist activity will be referred to as a subset model.

We considered 16,368 subset models using 1,820 chemicals from Judson et al. (2020) to identify the optimal assay battery to test for both agonism and antagonism. Although the NVS assays are no longer available, they were included in the analysis as proxies for future receptor-binding assays. The minimum criteria for a subset model to be considered for the optimal assay battery were as follows: sensitivity greater than 95% and specificity greater than 85% across all chemicals for either AR agonism or antagonism prediction by the subset model when compared against the prediction from the 14-assay model. This produces two sets of subset models, one for chemicals’ AR antagonism detection and the other for AR agonism detection. Then, we identified the union of assays for every pair of subset models from the two sets (i.e., AR agonism and antagonism). In the next step, we kept pairs of subset models with the minimum number of assays in the union for further analysis, and we removed pairs of subset models with a higher number of assays in the battery. This step is an optimization process that minimizes the number of assays needed for combined AR agonism and antagonism prioritization. In the final step, we identified the best subset models for AR antagonism and agonism prioritization. Because the optimal assay battery depends on the desired prioritization strategy, multiple options corresponding to different prioritization scenarios are discussed.

### 2.4 Evaluate prioritization options that leverage chemical clusters

#### 2.4.1 Identification of EDSP UoC clusters with potential AR active chemicals

Not all clusters contain AR active chemicals. We used two computational methods to estimate AR active chemicals: the 14-assay AR model and CoMPARA QSAR executed from the OPEn structure-activity/property Relationship App (OPERA) v2.7 suite (Mansouri et al., 2020). Additionally, we wanted to remove potentially volatile chemicals from consideration because obtaining accurate screening results from these chemicals can be difficult. Previously, EPA defined criteria for using the Henry’s law constant to categorize chemicals into “high,” “moderate,” “slight,” or “none” with regard to aqueous volatility within Resource Conservation and Recovery Act wastes (U.S. EPA, 1985). We used OPERA to calculate physico-chemical properties, including the Henry’s law constant, for the 6,166 EDSP chemicals with QSAR-ready SMILES strings that did not represent the chemical component of an unknown, variable composition, complex reaction products, or biological material (UVCB). Then, we compared the predicted Henry’s law constant (i.e., the air-water partition coefficient) from OPERA against the previously defined aqueous volatilization rate categories (U.S. EPA, 1985) and assigned each chemical to a volatilization category. After the categorization, chemicals with a volatility categorization of “high” or “moderate” were considered volatile and removed from the investigation.

We overlayed the 14-assay AR model and, separately, the CoMPARA QSAR predictions over the EDSP UoC clustering to identify clusters with potential AR activity. Clusters containing chemicals with possible AR agonist effects were marked as “agonist active clusters,” clusters containing chemicals with potential AR antagonist effects were labeled “antagonist active clusters,” clusters containing chemicals with both AR agonist and antagonist effects were marked as “both effect clusters,” and clusters in which chemicals did not show AR effects were labeled as “no effect clusters.” Predictions from CoMPARA and the 14-assay AR model are given side by side in the Supplementary Table S1.

#### 2.4.2 Multi-stage prioritization workflow

The multi-stage prioritization workflow is based on the observation that AR agonist chemicals are present in similar clusters. The method utilizes a 6-assay battery for the first stage. This battery includes a 5-assay AR antagonism model, which identifies AR antagonists with sensitivity >95% and specificity >85%. It also includes a 3-assay AR agonist model, which identifies AR agonists with sensitivity >70%. Although this sensitivity is below our minimum criteria, it still picks up ∼70% of the agonist chemicals and the clusters in which they are located. All identified AR agonist and antagonist chemicals in the first stage are candidates for further investigation. However, to boost agonist detection, the multi-stage prioritization workflow overlays positive AR agonists onto EDSP UoC clusters and identifies agonist-containing/enriched clusters. Then, a second prioritization of the chemicals within these clusters is performed using three additional assays, which, when combined with the six assays from the first stage, comprise a 9-assay battery (with sensitivity >95%). Finally, the best 9-assay subset model for AR agonist detection is used to identify positive AR agonists missed in the first run.

The results for the multi-stage workflow are based on a simulation study designed to approximate the expected results from a given submodel. For this simulation, the CoMPARA results are considered to be confirmed agonist and antagonist designations, and chemicals are sampled based on the sensitivity and specificity of the corresponding model. For example, the 3-assay agonist model contained within the optimal 6-assay battery has a sensitivity of 0.714, so we sampled this percentage of the chemicals with positive CoMPARA agonist predictions for Stage 1. The specificity of this 3-assay agonist model is 0.989, so we sampled 1.1% (1–0.989) of the chemicals with negative CoMPARA agonist predictions as the false positives for Stage 1. Based on the active chemicals (true positives and false positives) from Stage 1, the clusters are selected for Stage 2, and all chemicals from those clusters are sampled in Stage 2 using the sensitivity (0.96) and specificity (0.977) values associated with the 6-assay agonist model corresponding to the 9-assay battery.

## 3 Results and discussion

### 3.1 Comparison of the 14-assay AR model with the 11-assay AR model

Results from our analysis using the 14-assay model from (Judson et al., 2020) were compared with the original 11-assay model results from (Kleinstreuer et al., 2017) (Supplementary Table S2). Specifically, binarized activity calls based on the AUC values from each model were compared for 1,817 chemicals in common between the two datasets. The 14-assay model investigates potential AR antagonism or agonism on a set of 1,820 chemicals, whereas the original Kleinstreuer 11-assay model assesses 1,855 chemicals. Three chemicals, SSR 103800 (1075752-90-7, DTXSID1047364), SAR 150640 (433212-21-6, DTXSID4047389), and SSR 240612 (464930-42-5, DTXSID2047351), from the 14-assay model were not included in the original analysis using the Kleinstreuer 11-assay model.

We analyzed the number of chemicals for which the two AR models (Kleinstreuer 11-assay vs. 14-assay) agreed or disagreed on predictions (Table 1). In total, the results between the two analyses agree regarding antagonism activity for 1,733 out of 1,817 (>95%) chemicals and regarding agonism activity for 1,798 out of 1,817 (>98.9%) chemicals. The majority of the chemicals where the two models agree were predicted to be inactive, with 1,777 out of 1,798 chemicals predicted to be inactive in the agonist mode and 1,560 out of 1,733 chemicals predicted to be inactive in the antagonist mode. However, there are 84 out of 1,817 (4.6%) chemicals in the antagonist mode and 19 out of 1,817 (1%) in the agonist mode, for which the 14-assay AR model and Kleinstreuer 11-assay AR model disagree. Of these disagreements, the 14-assay AR model predicted antagonist activity for 74 out of 84 (88%) chemicals and agonist activity for 7 out of 19 (36.8%) chemicals, whereas the Kleinstreuer 11-assay model predicted inactivity. For the remaining disagreements, the Kleinstreuer 11-assay AR model predicted antagonist activity for 10 out of 84 (11.9%) chemicals and agonist activity for 12 out of 19 (63.2%) chemicals, whereas the 14-assay model predicted inactivity. This corresponded to a Matthews correlation coefficient of 0.79 for antagonist predictions and 0.68 for agonist predictions between the two models. The lower correlation for the agonists is not surprising, given the smaller number of agonists predicted by both models. In total, the 14-assay model is more sensitive with respect to antagonism, whereas the original 11-assay Kleinstreuer model predicts slightly more chemicals to be agonists.

Most disagreements correspond to antagonist predictions from the 14-assay model, where the original Kleinstreuer analysis predicted inactivity. Out of the 74 antagonist calls, the 14-assay battery included a hit call in the new antagonist assay (UPITT_HCI_U2OS_AR_TIF2_Nucleoli_Antagonist) for 70 chemicals, with an additional three chemicals having a hit call in the other new assay corresponding to nuclear translocation/coactivator interaction (UPITT_HCI_U2OS_AR_TIF2_Nucleoli_Agonist). This nuclear translocation/coactivator interaction assay is one of the steps in common between the agonist and antagonist pathways (Supplementary Table S3). Of the 73 chemicals with activity in one of the two new assays in the 14-assay model, the additional activity was responsible for the change in antagonist prediction for 63 chemicals.

**FIGURE 1:**
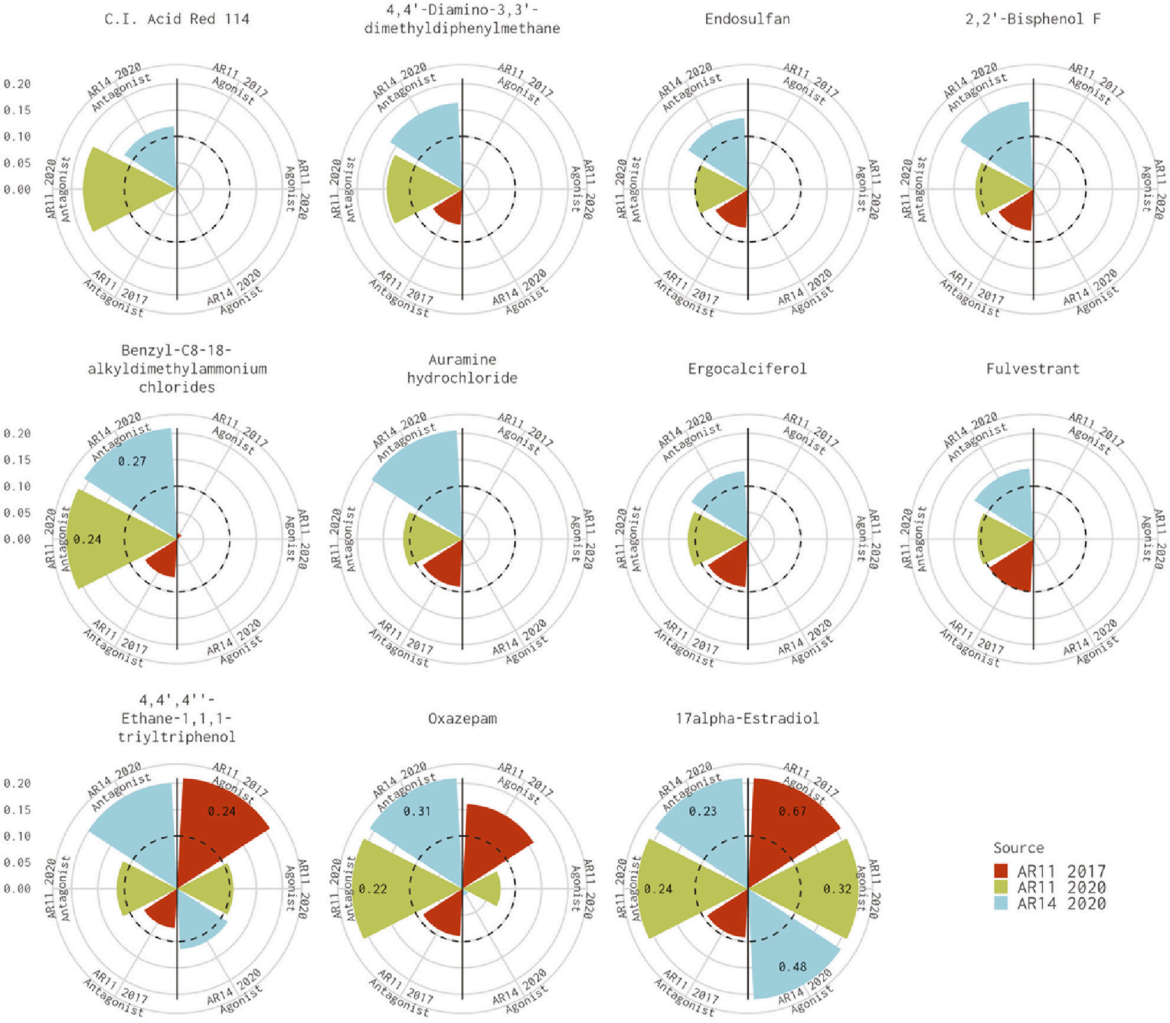
Comparison of antagonist AUC values from the Kleinstreuer and Judson models. These radial plots illustrate AUC values for chemicals where the Kleinstreuer 11-assay model (shown in red) did not meet the AUC threshold for antagonism, whereas the same 11-assay model (shown in green) or the 14-assay model (shown in blue) from the current analysis did meet the AUC threshold. The AUC threshold of 0.1 is shown by the dashed line. Each plot is limited to an AUC of 0.2 to increase legibility; AUCs exceeding the limit are labeled with the actual value of the AUC. The antagonist model results are on the left-hand side of each plot, and the agonist model results are on the right-hand side of each plot.

Figure 1 shows the AUC values for the remaining 11 chemicals where the 11-assay model from our analysis agreed with the 14-assay prediction and disagreed with the original Kleinstreuer prediction. There were six chemicals where the original 11-assay Kleinstreuer model AUC was slightly below the threshold (0.07<AUC≤0.1) and the current 11-assay model was just above the threshold (0.1<AUC<0.12). For the remaining five chemicals, the difference between the current 11-assay model and the original 11-assay Kleinstreuer model AUC was greater than the difference between the current 11- and 14-assay models, suggesting that the main difference is not primarily due to the two additional antagonist assays. The antagonist AUC from the 14-assay model was still higher than the 11-assay AUC for all chemicals except 17α-estradiol (57-91-0, DTXSID8022377), which was flagged as both an agonist and an antagonist by both the 11- and 14-assay models in the current analysis, and C.I. Acid Red 114 (6459-94-5, DTXSID8021224). The latter chemical was the only chemical that did not show activity in either of the two new antagonist assays.

Differences between the 11-assay model from our analysis and the original 11-assay Kleinstreuer model could be due to either the input data or alterations to the (pre-)processing introduced by (Judson et al., 2020) when expanding to the 14-assay model. Our analysis used data from ToxCast invitrodb v3.3, whereas the original Kleinstreuer analysis used data from invitrodb v2 (U.S. EPA, 2017), which was the active version in January 2017. There were many updates to the ToxCast data processing pipeline during this time, which could explain many of the discrepancies noted in our analysis (Filer et al., 2016; Sheffield et al., 2022).

In addition, the newer implementation by Judson et al. (2020) uses a different reference antagonist, mifepristone (84371-65-3, DTXSID5023322), because the previous reference chemical [hydroxyflutamide (52806-53-8, DTXSID8033562)] was not tested in all the new assays (Judson et al., 2020).

**FIGURE 2:**
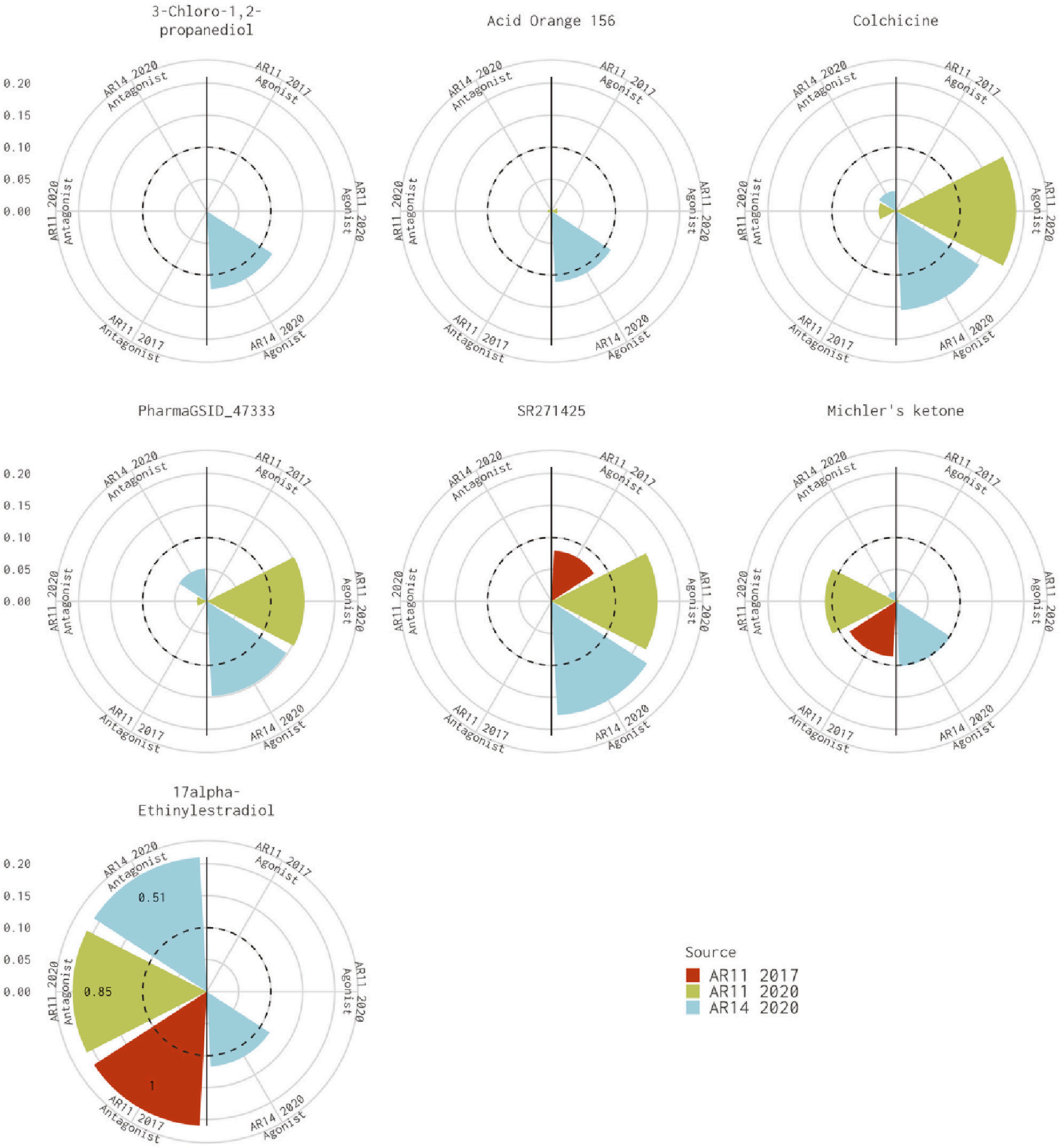
Comparison of agonist AUC values from the Kleinstreuer and Judson models. These radial plots illustrate AUC values for chemicals where the Kleinstreuer 11-assay model (shown in red) did not meet the AUC threshold for agonism, whereas the same 11-assay model (shown in green) or the 14-assay model (shown in blue) from the current analysis did meet the AUC threshold. The AUC threshold of 0.1 is shown by the dashed line. Each plot is limited to an AUC of 0.2 to increase legibility; AUCs exceeding the limit are labeled with the actual value of the AUC. The antagonist model results are on the left-hand side of each plot, and the agonist model results are on the right-hand side of each plot.

There are seven chemicals where the 14-assay AR model predicts agonist activity but the original 11-assay Kleinstreuer AR model predicts inactivity (Figure 2). The 14-assay model has two additional assays in the agonist pathway: UPITT_HCI_U2OS_AR_TIF2_Nucleoli_Agonist assay and ACEA_AR_agonist_80h. In this case, activity in the ACEA_AR_agonist_80h was responsible for the difference for four chemicals (Supplementary Table S4), whereas the remaining three chemicals also have agonist calls with the 11-assay model in our current analysis (Figure 2) and are, therefore, likely due to differences in the input data or normalization.

**FIGURE 3:**
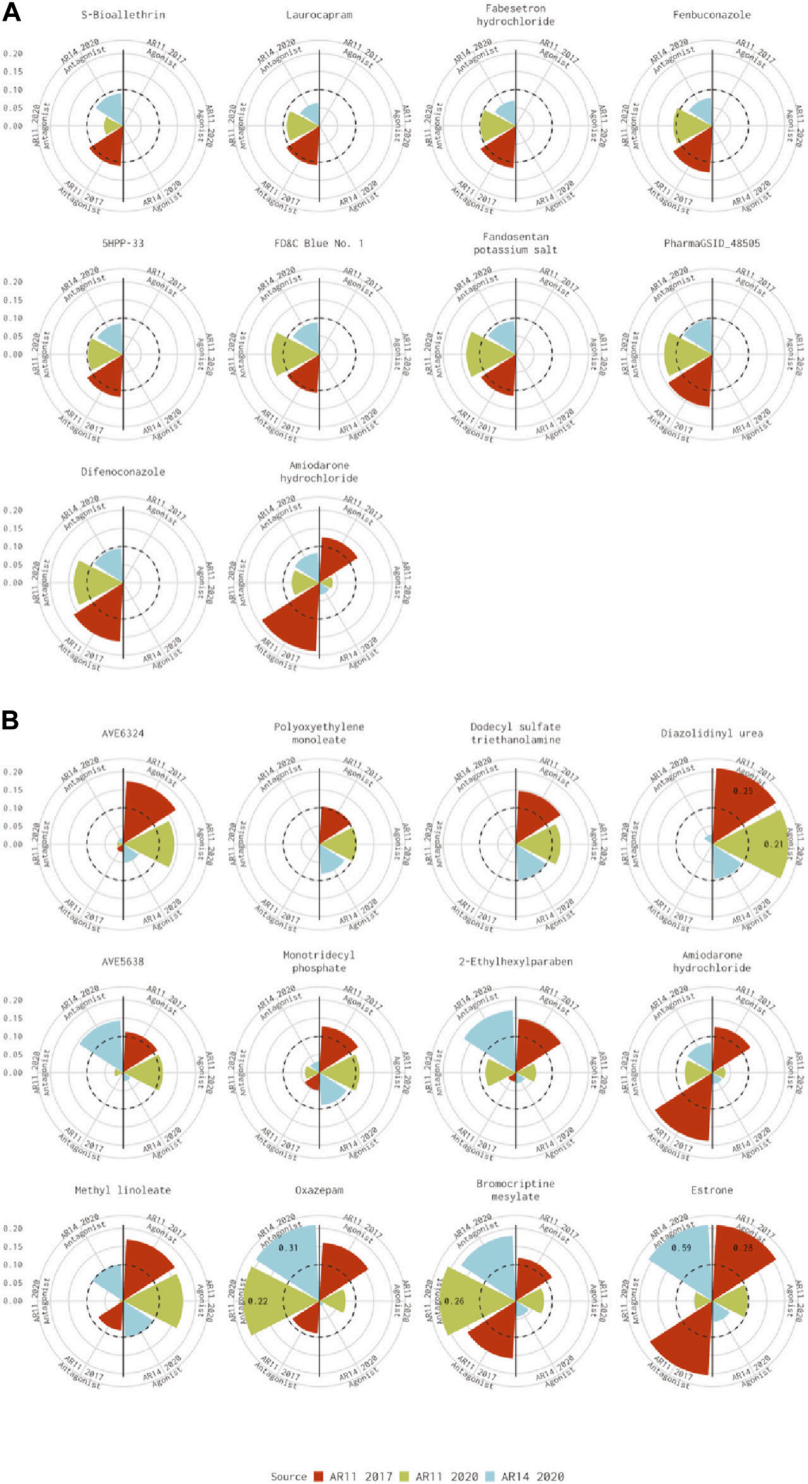
Evaluation of active chemicals from the Kleinstreuer model that were inactive in the Judson model. These radial plots illustrate AUC values for chemicals where the Kleinstreuer 11-assay model (shown in red) did meet the AUC threshold for **(A)** antagonism and **(B)** agonism, whereas the same 11-assay model (shown in green) or the 14-assay model (shown in blue) from the current analysis did not meet the AUC threshold. The AUC threshold of 0.1 is shown by the dashed line. Each plot is limited to an AUC of 0.2 to increase legibility; AUCs exceeding the limit are labeled with the actual value of the AUC. The antagonist model results are on the left-hand side of each plot, and the agonist model results are on the right-hand side of each plot. Amiodarone hydrochloride (DTXSID7037185) is included in both panels.

For the 10 chemicals predicted to be antagonists by Kleinstreuer et al. (2017) and inactive in the current analysis, the 14-assay model AUC was just under the threshold (0.08<AUC<0.1), or the original 11-assay Kleinstreuer model AUC was close to the threshold (0.1<AUC<0.12) for all chemicals except fenbuconazole [DTXSID8032548 (Figure 3A)]. For the 12 chemicals predicted to be agonists by Kleinstreuer et al. (2017) and inactive in the 14-assay model, the 14-assay model predicted antagonism for six chemicals, including two where the original 11-assay Kleinstreuer model predicted both agonism and antagonism (Figure 3B). The 11-assay model from the current analysis agreed with the original 11-assay Kleinstreuer model for five additional chemicals, suggesting that the addition of the three new assays was responsible rather than the impacts from input data or normalization. For four of these chemicals, the 14-assay model agonist AUC was close to the threshold (0.08<AUC<0.1). The last chemical from this set was AVE6324 (DTXSID0047377), which showed agonist activity in both 11-assay models, but the 14-assay agonist AUC was 0.05. The final chemical was amiodarone hydrochloride, which was predicted to have both agonist and antagonist activity by the original 11-assay Kleinstreuer model but predicted to be inactive by both the 11- and 14-assay models in the current analysis.

The lowest 25th percentile of AUC values associated with active chemicals was approximately 0.14 (0.139 agonist, 0.143 antagonist) for the 14-assay model and slightly higher (0.148 agonist, 0.162 antagonist) for the Kleinstreuer model. Of all the discrepancies where the Kleinstreuer model predicted activity and the 14-assay model predicted inactivity, only six were above this threshold (three antagonist and three agonist predictions), and in four of those cases, the AUC from the 14-assay model was greater than 0.09. The majority of the false positives from both models are expected to fall within that lower quartile, which makes it impossible at this stage to interpret the results in this range. Interpretation of the agonist results is further complicated by the fact that none of the reference chemicals to date have an AUC lower than 0.285 in either model. As more data are collected for AR agonists, predictions for these very weak agonists should improve. In the meantime, chemicals that are likely to show strong AR activity should be flagged by either model.

### 3.2 Structural diversity of AR agonists and antagonists

Of 1,820 chemicals in Judson et al. (2020), the 14-assay model predicted 244 AR antagonists and 22 AR agonists. Six chemicals [tannic acid (1401-55-4, DTXSID2026076), 4,4′,4″-Ethane-1,1,1-triyltriphenol (27955-94-8, DTXSID2037712), melengestrol acetate (2919-66-6, DTXSID5048184), cyproterone acetate (427-51-0, DTXSID5020366), 17α-ethinylestradiol (57-63-6, DTXSID5020576), and 17α-estradiol (57-91-0, DTXSID8022377)] were predicted to produce both AR agonism and antagonism. For the remaining 1,548 chemicals, the 14-assay AR model predicted no AR effect.

The (Nelms et al., 2023) cluster assignments consist of 826 clusters with 7,954 EDSP UoC DTXSIDs. From the list of 1,820 AR model chemicals in Judson et al. (2020), 69 did not have SMILES strings in the CompTox dashboard; therefore, they were not assigned to clusters. The remaining 1,751 chemicals with structural information were assigned to 561 clusters based on structural similarity, from which 371 clusters had more than one AR model chemical. Out of 272 active chemicals, 259 (5 out of 6 agonists/antagonists, 22 out of 22 agonists, and 232 out of 244 antagonists) had the structural information required for a cluster assignment.

AR active chemicals based upon the 14-assay model are distributed across 136 clusters. In 130 clusters, we identified chemicals predicted as active AR antagonists by the 14-assay AR model (Table 2). In 10 clusters, we identified chemicals predicted as active AR agonists by the 14-assay AR model. In clusters 439, 534, and 789, we identified five chemicals predicted to have both effects. Tannic acid (1401-55-4, DTXSID2026076), which would be the sixth chemical predicted as both an agonist and antagonist, is a substance with a UVCB and hence no discrete chemical structure; as such, it was not part of cluster analysis. No AR antagonist or agonist effect was predicted for chemicals present in 425 clusters (Table 2).

**TABLE 2:** Summary of AR agonists and antagonists and the clusters containing them.

		Agonism + antagonism^a^	Only		Inactives (no active chemicals in cluster)
			Antagonism^a^	Agonism	
Active chemicals based on the 14-assay model	Number of clusters	3	130	10	531 (425)
	Number of chemicals	5	232	22	1,492 (1,103)
Active chemicals based on CoMPARA	Number of clusters	33	263	20	619 (382)
	Number of chemicals	93	817	40	3,283 (2,095)

Readers should refer to Supplementary Table S1 for chemical predictions and cluster assignments. Note that a single cluster may be counted in more than one column.

^a^ CoMPARA results exclude two chemicals for which the agonist prediction was not applicable.

**FIGURE 4:**
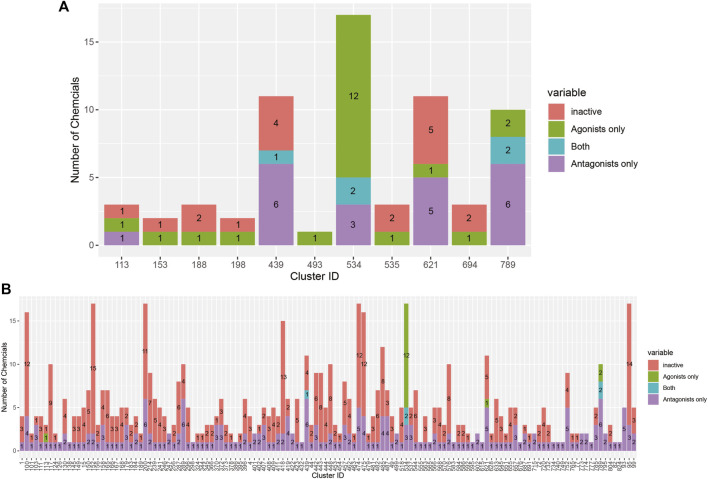
Bar chart illustrating the results from the 14-assay AR model for chemicals in clusters containing at least one agonist **(A)** or antagonist **(B)**. Chemicals in each cluster are categorized by whether they are predicted to be an agonist, antagonist, both agonist and antagonist, or inactive.

The breakdown of all clusters containing at least one active chemical is shown in Figure 4. The majority of the agonists fall within clusters that also contain antagonists (Figure 4A). Each of the six agonists that are not clustered with an antagonist falls within a small cluster with only one active chemical, suggesting that some of them may be false positives. Conversely, most of the antagonist clusters do not contain any agonist chemicals (Figure 4B). Here again, however, many of the clusters contain a single active chemical. The structural similarities between the agonists and antagonists may also explain why around 20% of the agonists were predicted to be both agonists and antagonists (Table 2). The increased structural diversity for antagonism is not surprising because these chemicals need only prevent the AR from activating, which allows for a wide array of receptor conformations, whereas an agonist must force the receptor into an active conformation.

In addition, we submitted 7,954 EDSP DTXSIDs to CoMPARA QSAR for AR activity estimations and obtained predictions for AR agonism and antagonism. For molecules that did not have appropriate structural information, “NaN” was returned with the explanation of the error. Furthermore, CoMPARA did not return AR agonism and antagonism predictions for C.I. acid red 186 (DTXSID2044688) and octasodium 4,4′-bis{[4-chloro-6-({6-[(1,5-disulfonato-2-naphthalenyl)diazenyl]-5-hydroxy-7-sulfonato-2-naphthalenyl}amino)-1,3,5-triazin-2-yl]amino}[biphenyl]-2,2′-disulfonate (DTXSID00893611). Also, the result was missing for AR agonism for fluorescein (DTXSID0038887) and fluorescein sodium (DTXSID9025328). After removing volatile substances, which would be difficult to screen, we obtained a set of 4,235 EDSP molecules with CoMPARA results.

**FIGURE 5:**
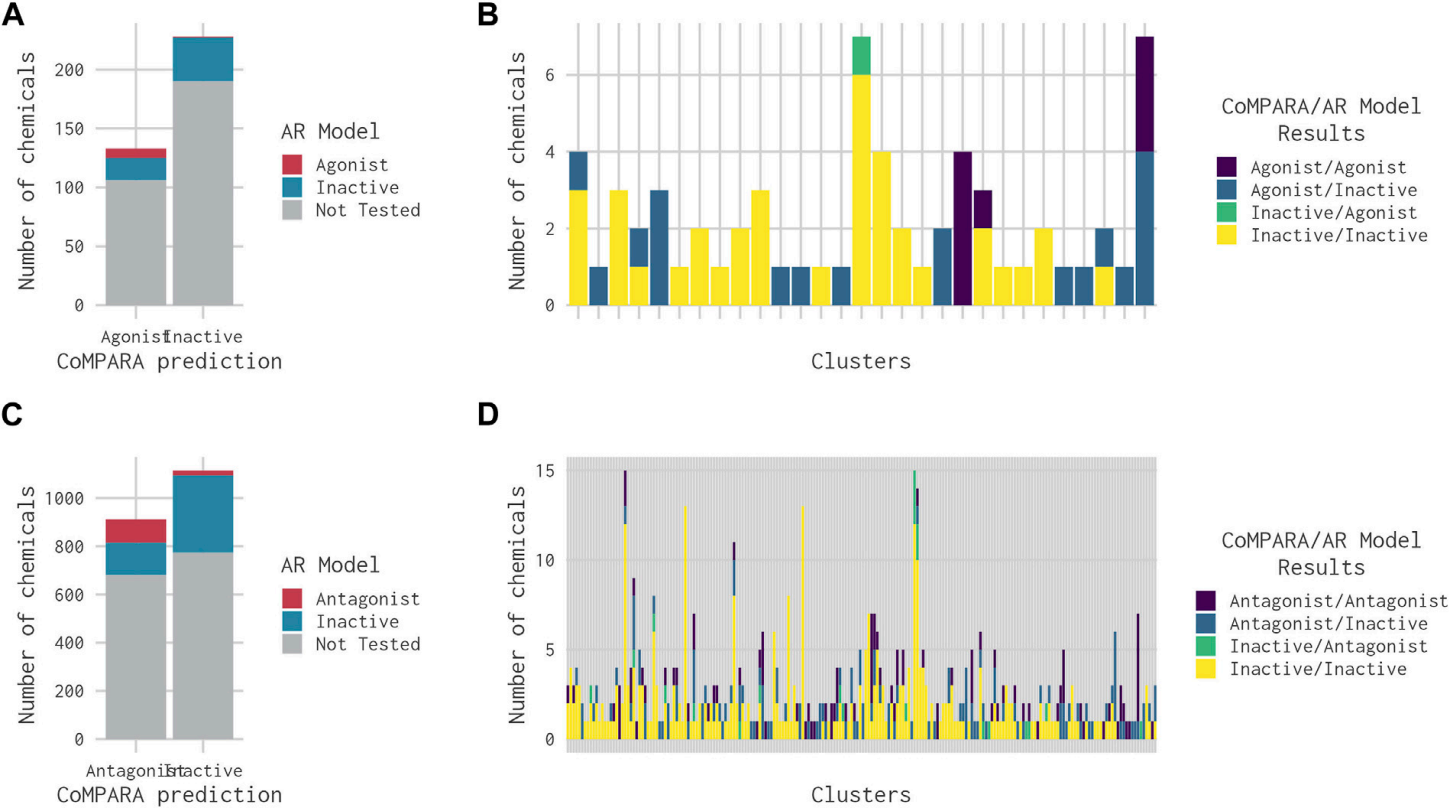
Bar charts comparing the results from the 14-assay AR model to the predictions from the CoMPARA QSAR model for chemicals in clusters with an (ant)agonist prediction in at least one of the models. **(A)** Overall comparison of CoMPARA and AR model agonist predictions. **(B)** Comparison of CoMPARA and AR model agonist predictions by cluster. **(C)** Overall comparison of CoMPARA and AR model antagonist predictions. **(D)** Comparison of CoMPARA and AR model antagonist predictions by cluster. Predictions for all chemicals can be found in Supplementary Table S1.

CoMPARA predicted 952 AR active chemicals, which were assigned to 140 clusters. AR agonist chemicals (133) were contained within 53 clusters (Table 2), of which 40 molecules were predicted with only an AR agonist effect, and 93 chemicals were predicted with both AR agonist and AR antagonist effects. Comparison of the CoMPARA results with those from the 14-assay AR model shows that the CoMPARA predictions are conservative, with the majority of the discrepancies being false positives (Figures 5A, C). In total, there were 22 cases (1 agonist, 21 antagonists) where the 14-assay model predicted activity but CoMPARA did not, compared with 152 (19 agonists, 133 antagonists) where CoMPARA predicted activity but the 14-assay model did not. For agonists, the agreement or disagreement between the models was mostly consistent within a cluster, whereas with antagonists, the results were mixed (Figures 5B, D).

### 3.3 Key assays for high-performing subset models

A total of 16,368 subset models were generated using the “ARminassaymodel” package. We identified 537 subset models with AR antagonist specificity >85% and sensitivity >95% across 1,820 chemicals. In addition, for AR agonist prediction, 109 subset models were identified with specificity >85% and sensitivity >95% across 1,820 chemicals. These are the subset models we considered acceptable for AR agonist and antagonist prediction.

**FIGURE 6:**
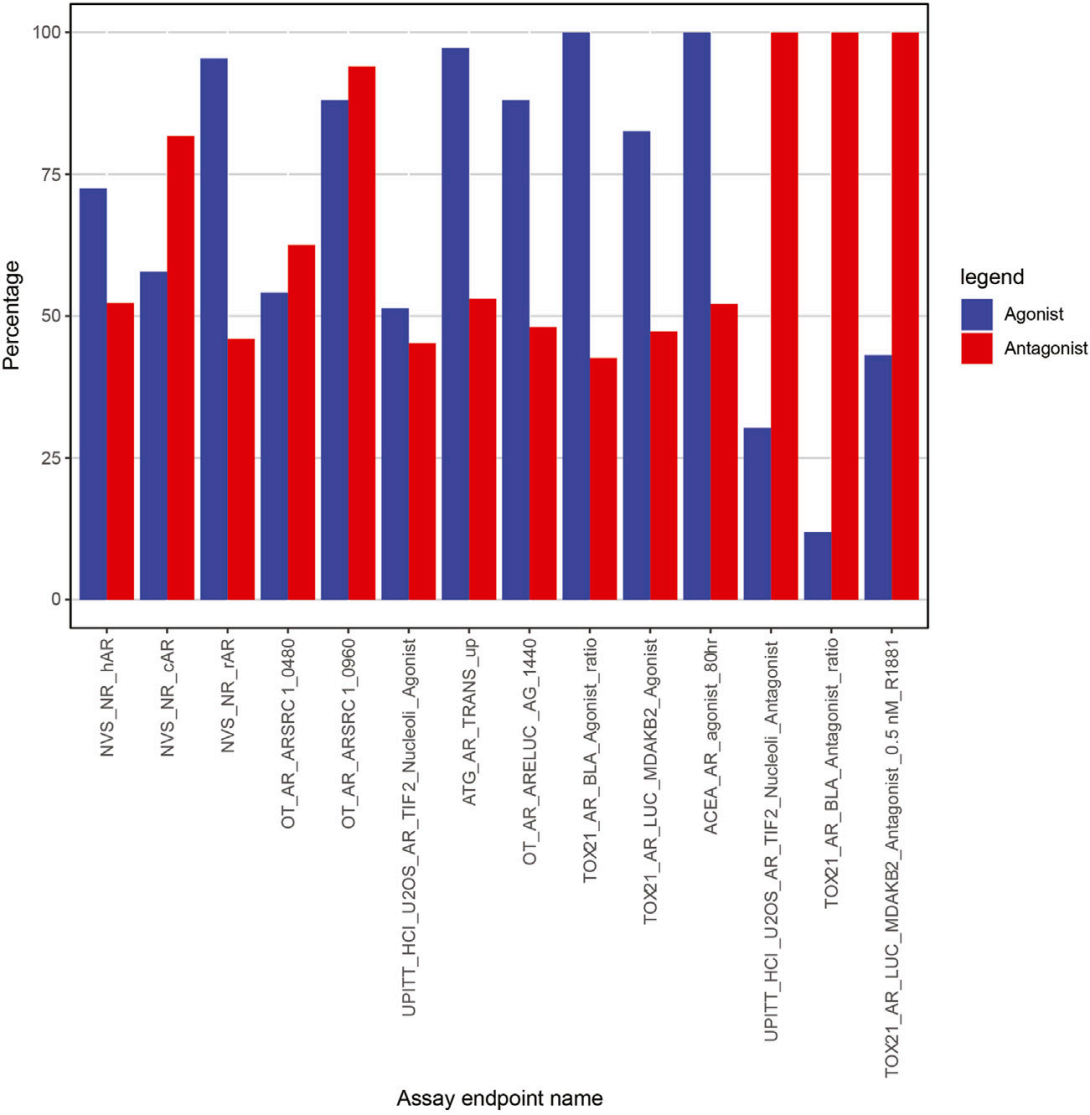
Percentage of assays in subset models with specificity >85% and sensitivity >95%.

To understand the importance of assays in subset model predictivity, we calculated their occurrence in the 537 AR antagonist subset models and 109 AR agonist subset models that satisfied the specificity and sensitivity criteria (Figure 6). For example, TOX21_AR_LUC_MDAKB2_Antagonist_0.5 nM_R1881 (far right bar in Figure 6) is present in 100% of the 537 antagonist subset models that meet our minimum sensitivity and specificity criteria. However, the same assay is present in only 43.12% (47 out of 109) of the subset models for agonism AR detection.

Not surprisingly, all three antagonist-specific assays (UPITT_HCI_U2OS_AR_TIF2_Nucleoli_Antagonist, TOX21_AR_BLA_Antagonist_ratio, and TOX21_AR_LUC_MDAKB2_Antagonist_0.5 nM_R1881) are present in 100% of the high-performing submodels for antagonism (Figure 6), so these would be strong candidates for inclusion in any prioritization strategy focused on AR antagonism. The other two assays that are present in a large number of the successful AR antagonism subset models are OT_AR_ARSRC1_0960 (>94%) and NVS_NR_cAR (>81%). Of note, OT_AR_ARSRC1_0960 is one of the nuclear translocation/coactivator interaction assays, and NVS_NR_cAR is one of the AR-binding assays. Together, these two assays cover the different biological steps that are common to both agonism and antagonism, so their inclusion adds additional plausibility to our data-driven selection strategy.

There were two assays (TOX21_AR_BLA_Agonist_ratio and ACEA_AR_agonist_80h) in 100% of subset models for AR agonist prioritization (Figure 6). Without these two assays, none of the subset models meet our AR agonist sensitivity and specificity criteria. As expected, both assays are specific to the agonist-only set of assays. The next most-prevalent assay within the subset models for AR agonism (ATG_AR_TRANS_up, 97.25%) was also an agonist-only assay. Collectively, these top three assays cover the three different biological components associated with agonism (RNA transcription, protein production, cell proliferation). As seen with the antagonists, there was one highly prevalent assay from both the AR binding set (NVS_NR_rAR, 95.41%) and the nuclear translocation/coactivator interaction set (OT_AR_ARSRC1_0960, 88.07%). Although our data-driven approach aids in selecting the optimal assay from each biologically equivalent set, the results are entirely consistent with what would be expected from an expert-driven approach focused on comprehensively covering the biological steps.

A complementary analysis is to check the sensitivity of the 13-assay subset model after removal of a single assay to determine the impact of that individual assay. If an assay is essential for detecting AR active chemicals, its removal will substantially drop the sensitivity metric in the resulting 13-assay model; conversely, if an assay is less critical, the sensitivity will remain high or close to 100%. Table 3 shows the maximum sensitivity of subset models without 1 of 14 assays. Subset models without any one of the antagonism-specific assays (UPITT_HCI_U2OS_AR_TIF2_Nucleoli_Antagonist, TOX21_AR_BLA_Antagonist_ratio, or TOX21_AR_LUC_MDAKB2_Antagonist_0.5 nM_R1881) cannot reach our minimum sensitivity threshold of 95%, with maximum antagonist sensitivities of 83.6%, 93.2%, and 92%, respectively.

**TABLE 3:** Maximum sensitivity performance of all remaining subset models after an assay exclusion.

Assay ID	Absence of assay endpoint	Max sensitivity	
		Antagonist	Agonist
A1, A2, and A3	All receptor-binding assays (antagonist and agonist pathway)	95.6	89.3
A1	Receptor-binding assay NVS_NR_hAR (antagonist and agonist pathway)	97.2	96.4
A2	Receptor-binding assay NVS_NR_cAR (antagonist and agonist pathway)	97.2	100
A3	Receptor-binding assay NVS_NR_rAR (antagonist and agonist pathway)	98	96.4
A4	Coregulator recruitment assay OT_AR_ARSRC1_0480 (antagonist and agonist pathway)	97.6	100
A5	Coregulator recruitment assay OT_AR_ARSRC1_0960 (antagonist and agonist pathway)	97.2	96.4
A6	Nuclear translocation assay UPITT_HCI_U2OS_AR_TIF2_Nucleoli_Agonist (antagonist and agonist pathway)	98	100
A7	RNA reporter gene assays (ATG_AR_TRANS_up) (agonist pathway)	100	96.4
A8	Reporter gene assay OT_AR_ARELUC_AG_1440 (agonist pathway)	100	96.4
A9	Reporter gene assay TOX21_AR_BLA_Agonist_ratio (agonist pathway)	100	89.3
A10	Reporter gene assay TOX21_AR_LUC_MDAKB2_Agonist (agonist pathway)	100	96.4
A11	Real-time impedance assay (ACEA_AR_agonist_80h) (agonist pathway)	100	89.3
A12	Nuclear translocation assay UPITT_HCI_U2OS_AR_TIF2_Nucleoli_Antagonist (antagonist pathway)	83.6	100
A13	Reporter gene assay TOX21_AR_BLA_Antagonist_ratio (antagonist pathway)	93.2	100
A14	Reporter gene assay TOX21_AR_LUC_MDAKB2_Antagonist_0.5 nM_R1881 (antagonist pathway)	92	100

The “Max sensitivity” columns consider all remaining subset models.

AR agonist sensitivity across all subset models is influenced by only the TOX21_AR_BLA_Agonist_ratio and ACEA_AR_agonist_80h assays. If either of these two assays is absent, the remaining subset models will have a maximum agonist sensitivity of 89.3% (Table 3). Both assays are exclusive to the agonist pathway in the AR model. Although removing no individual receptor-binding assay greatly impacts agonist sensitivity (i.e., sensitivity remains ≥95%), the elimination of all three binding assays reduces agonist sensitivity to 89.3%, which indicates that receptor binding is essential for meeting the agonist prioritization criteria. The impact on antagonism prediction is not as great, with the removal of all three binding assays still having a sensitivity greater than 95%.

### 3.4 Minimal assay requirements for prioritization based upon agonism and antagonism

From Judson et al. (2020), the smallest subset model that could simultaneously predict both agonism and antagonism consisted of 13 assays, with two different models satisfying the minimum criteria: A11111011111111 and A11101111111111. From a practical perspective, however, the difference between running 13 or 14 assays is negligible. As shown in the previous section, the assays important for agonist vs. antagonist prediction are largely distinct based on the original model design. As a result, the inclusion of agonist-specific assays when modeling antagonism and *vice versa* likely reduces the performance of the model via the inclusion of non-informative assays. Therefore, we looked for the smallest assay battery that contains at least one subset model for agonism and one subset model for antagonism that meet our minimum sensitivity and specificity criteria for both modes.

For every pair of subset models where one predicts agonism and the other predicts antagonism and both meet our minimum criteria, we calculated the number of assays in the union. In total, 58,533 pairs of subset models were evaluated and the difference in assay content between the models was calculated (i.e., the Hamming distance). The result is available in Supplementary Table S5. A battery of nine assays is the smallest set of assays from which subset models can be created to simultaneously satisfy our defined performance criteria for the chemicals’ AR agonism and antagonism detection.

Table 4 shows the different pairs of subset models that combine to give nine assays total. There is a single subset model for AR agonism (A00101011101000) and 13 different AR antagonism subset models. The AR agonism subset model contained six assays and excluded the three antagonism assays. In contrast, all antagonism models included the three antagonism-specific assays combined with anywhere from two to six additional assays that were also included in the 6-assay agonist model. The sensitivity for all antagonist models was 0.952, and the specificity ranged from 0.934 to 0.959 (Table 4). The fact all 13 antagonist subset models chosen had the same sensitivity appears to be driven (at least in part) by the threshold criteria where a subset model was required to have a sensitivity of at least 95%, with each of these antagonist subset models having the minimum number of chemicals with a true positive prediction to have a sensitivity greater than 95%. For example, each of these models have 238 chemicals with a true positive prediction and 12 chemicals with a false negative (238/250 = 0.952). Although a single model to predict agonism and antagonism eliminates only 7% (1 out of 14) of the required assays, the use of two subset models tailored to predict either agonism or antagonism can reduce the resources required by 36% (5 out of 14) while still meeting the minimum sensitivity and specificity criteria.

**TABLE 4:** Union of assays between antagonist and agonist AR subset models. The sensitivity of 0.952 across all antagonist subset models appears to be driven by the threshold we placed on the minimum sensitivity criterion (i.e., >95%). NB: the sensitivity and specificity for the single AR agonist model used here is 0.964 and 0.977, respectively.

Subset model		Assays in union (#)	Hamming distance	Antagonist subset model	
Agonist	Antagonist			Sensitivity	Specificity
A00101011101000	A00101000000111	9	7	0.952	0.952
A00101011101000	A00001010000111	9	7	0.952	0.934
A00101011101000	A00001000001111	9	7	0.952	0.934
A00101011101000	A00001001000111	9	7	0.952	0.934
A00101011101000	A00101000001111	9	6	0.952	0.955
A00101011101000	A00101010000111	9	6	0.952	0.954
A00101011101000	A00001001001111	9	6	0.952	0.936
A00101011101000	A00001011000111	9	6	0.952	0.936
A00101011101000	A00001010001111	9	6	0.952	0.934
A00101011101000	A00101010001111	9	5	0.952	0.955
A00101011101000	A00001011001111	9	5	0.952	0.936
A00101011101000	A00101010101111	9	4	0.952	0.957
A00101011101000	A00101011101111	9	3	0.952	0.959

As we saw when looking at the key assays, the subset models corresponding to the 9-assay battery tend to couple individual assays from the two shared biological events with multiple assays specifically targeting agonism or antagonism. For example, the agonist assay contains one AR-binding assay, one nuclear translocation/coactivator interaction assay, and four out of five agonist-specific assays. Conversely, the antagonism subset models all included the three antagonist-specific assays and at least one assay for AR binding or nuclear translocation/coactivator interaction. Once again, our data-driven approach is entirely consistent with the biology underlying the different assays.

### 3.5 Optimizing the use of high-throughput assays for AR prioritization

To simultaneously achieve specificity >85% and sensitivity >95% for both antagonist and agonist predictions requires a 9-assay battery, but as few as five assays can achieve these minimum criteria for predicting antagonism. Because almost 90% of the active calls from the 14-assay model are antagonist predictions, the five assays important for predicting antagonism are much more valuable than the four needed to meet the minimum sensitivity threshold for agonism. Given this, are there different prioritization scenarios that could further reduce the resources required for AR activity predictions?

We considered all possible assay batteries of five to eight total assays that include an antagonism subset model meeting our minimum criteria (Table 4). Table 5 shows the best-performing pairs of subset models and highlights the maximum agonist sensitivity that can be achieved with fewer than nine total assays in the battery. Because all antagonist submodels meeting the minimum criteria include all three antagonist-specific assays, this leaves anywhere from two to five agonist-relevant assays in the battery. The addition of one assay to the five needed for acceptable antagonist predictions results in a 6-assay battery, from which a 5-assay AR antagonist model (antagonist sensitivity >95%) and a 3-assay AR agonist model (agonist sensitivity 71%) can be used for predicting potential AR activity. To improve performance further requires eight assays, which is not substantially different from the 9-assay battery that meets the minimum criteria.

**TABLE 5:** Minimal assay batteries containing a subset model meeting the minimal criteria for antagonism, which also contains the best-performing agonist subset model.

Assay battery	Model		Percentage (%)			
			Antagonists		Agonists	
	Antagonist	Agonist	Sensitivity	Specificity	Sensitivity	Specificity
5 assays	5 assays A00001000001111	2 assays A00000000001001	>95	>93	64.30	92.60
6 assays	6 assays A00001011000111	3 assays A00001011000000	>95	>93	71.40	98.90
7 assays	7 assays A00001011001111	5 assays A00001011001001	>95	>93	71.40	99.00
8 assays	8 assays A00101010101111	7 assays A00101010101101	>95	>95	85	99
9 assays	9 assays A00101011101111	6 assays A00101011101000	>95	>95	96	97.70

If we assume that 90% of the active chemicals are antagonists and further assume 95% sensitivity for antagonism and 70% sensitivity for agonism, then our 6-assay battery could provide an overall sensitivity of ∼92.5% (95% * 0.9 + 70% * 0.1). Because the 6-assay battery reduces the resources required by a third compared with the 9-assay battery, this small decrease in overall sensitivity is offset by a substantial savings in resources. Overall, the 6-assay battery would reduce the total resources required by 57% (8 out of 14) while missing our overall minimum sensitivity threshold by 2.5%.

**FIGURE 7:**
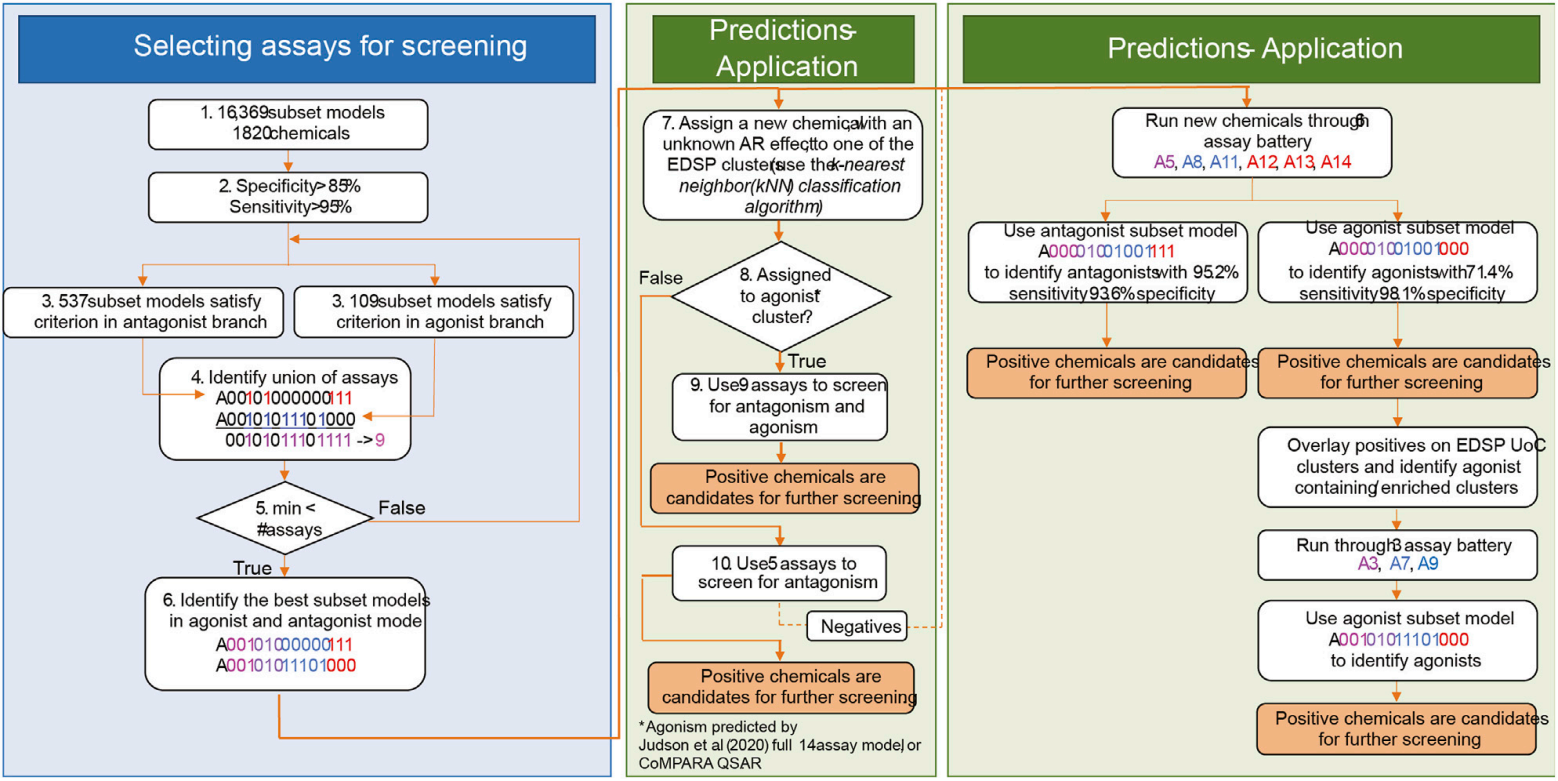
General approach to (1) selection of assays for AR prioritization and (2) application. The selection of assays for AR testing is shown in the blue box. In step 4, color schema and assay nomenclature follow Figure 1 in Judson, Houck et al. (2020). Assays identified in the union break out according to the assay design. Assays in magenta (A3 and A5) are the upstream assays present in both modes. Assays in blue (agonism-specific A7, A8, A9, and A11) are good in the detection of agonism effects. Assays represented in red (antagonism-specific A12, A13, and A14) are good for the detection of antagonist effects. Step 5 is an optimization step that minimizes the number of required assays for AR testing. The middle green box shows the selection of assays for chemical testing based on information from cheminformatics tools. In step 8, the 14-assay AR model and CoMPARA QSAR were used to estimate chemicals’ AR agonism. These chemicals were assigned to the clusters. Clusters that contain AR agonists are referred to as “agonist clusters.” The right green panel proposes an approach to boost the detection of agonists by performing two rounds of testing. We refer to this approach as multi-stage testing.

There is potential for further performance improvement by adjusting the testing strategy for chemicals that are more or less likely to be AR agonists (Figure 7). One approach would be to use the full set of nine assays for chemicals that are more likely to be agonists and the 5-assay battery for all other chemicals (middle box in Figure 7). Across the 1,820 substances from Judson et al. (2020), there are 11 clusters in which some chemicals are predicted to be AR agonists. If CoMPARA predictions are used, 46 clusters contain at least one predicted agonist. When considering all clusters having an agonist prediction from either model, there are 50 clusters containing 387 chemicals. In this scenario, one would use the 5-assay model for all substances within clusters that do not contain any chemical with an agonist prediction. Meanwhile, the 9-assay battery would be used for chemicals assigned to a cluster where at least one chemical has an agonist prediction.

An alternative approach would be to set up a two-step prioritization strategy where all chemicals are tested using the 6-assay battery identified earlier (right box in Figure 7). This step should pick up ∼70% of the real agonists, including those in clusters not covered by the original 1,820 substances tested with the 14-assay battery. A second round of prioritization with the three additional assays from the 9-assay battery could then be performed on all chemicals contained in clusters where an agonist was identified in the first stage. Both of these options would be expected to increase the overall sensitivity above the 92.5% expected with the 6-assay battery alone while still substantially decreasing the resources required to run all chemicals through the 9-assay model.

Because the last two options involve testing subsets of chemicals through different numbers of assays, the only way to compare the options is by considering a hypothetical testing scenario and comparing the total number of chemical-assay pairs (i.e., Stage 1 chemicals * Stage 1 assays + Stage 2 chemicals * Stage 2 assays). All options are summarized in Table 6 using the 4,235 chemicals having CoMPARA predictions as the basis for the calculations. For the first three options, this is simply the number of chemicals multiplied by the number of assays in the battery (i.e., no Stage 2 chemicals or assays).

**TABLE 6:** Summary of resources required and anticipated performance of different testing scenarios.

Testing scenario	Model(s)		Chemical-assay pairs	Percentage (%)	
	Antagonist	Agonist		Maximum	Overall sensitivity
13 assays (2 possible batteries): antagonist model = agonist model	A11111011111111, A11101111111111	A11111011111111, A11101111111111	55,055	93	>95
9 assay battery: 9-assay antagonist model, 6-assay agonist model	A00101011101111	A00101011101000	38,115	64	>95
6 assay battery: 5-assay antagonist model, 3-assay agonist model	A00001000001111	A00001011000000	25,410	43	∼92.5
Cluster-based testing: 9-assay battery for 387 chemicals from 50 agonist-containing clusters, 5-assay battery for remaining 3,848 chemicals	A00101011101111, A00001000001111	A00101011101000, A00000000001001	22,723	38	Unknown
Multi-stage testing: 6-assay battery for stage 1, 9-assay battery for stage 2	A00001000001111, A00101011101111	A00001011000000, A00101011101000	<28,400	48	∼95

The number of chemical-assay pairs is based upon hypothetical testing of 4,235 compounds using the testing scenario described in column 1. Rows 1–3 are the product of the number of assays in the battery and the number of compounds (4,235). The last two rows are estimated via simulation as described in the text. The computational models used to predict antagonism and agonism are in columns 2 and 3. Note that not all assays from the battery are necessarily included in each model. The percentage in column 5 is based upon testing 4,235 compounds through all 14 AR assays from the full Judson model. The overall sensitivity is estimated as described in the text.

For the cluster-based prioritization strategy, 50 clusters contain at least one chemical predicted to be an agonist either by CoMPARA or the 14-assay model. The 387 chemicals contained within those clusters are multiplied by nine assays, whereas the remaining 3,848 chemicals are multiplied by the 5-assay battery. This reduces the total resources by an additional 5% compared with testing all chemicals via the 6-assay battery. Because the overall sensitivity for this option is dependent on the accuracy of the existing agonist predictions, there is no simple way to estimate it. However, because the CoMPARA predictions seem to have a high negative predictive value for positives from the 14-assay model, this could represent a promising approach and would result in the fewest resources compared with all other options.

For the multi-stage prioritization strategy, the total number of chemical-assay pairs was estimated by simulating the staged testing procedure for the agonist chemicals and taking the average of 1,000 runs. The sensitivity and specificity for each stage are assumed to match those provided in Table 5 for the 6- and 9-assay batteries (i.e., Stage 1 is assumed to match the 3-assay agonist model, and Stage 2 is assumed to match the 6-assay agonist model). The mean number of chemicals that carried forward into the second stage was 988 (median = 986, range = 810–1,174), so the total number of chemical-assay pairs was calculated as six assays * 4,235 + three assays * 988. The agonist predictions were simulated by sampling from the CoMPARA dataset using the sensitivity and specificity values from Table 5. The average sensitivity for agonist detection in this simulation was 0.936 (median = 0.940, range = 0.872–1.0). The overall sensitivity was then calculated to be ∼95% (95 * 0.9 + 93.6 * 0.1).

By reducing the resources required for the initial testing, it is possible to test all chemicals more quickly and efficiently. Because the agonists and antagonists that are missed by the smaller subset models tend to have weaker effects, any compounds missed in the first round of testing would be highly unlikely to be prioritized for subsequent, labor-intensive screening steps in the near term. With the additional data collected from the initial testing, the cheminformatics approaches will become better able to predict these weaker agonists, which could either eliminate the need for further high-throughput testing or aid in the design of more efficient prioritization strategies for identifying the weaker agonists and antagonists.

### 3.6 Performance of subset models without receptor-binding assays for detection of AR antagonism and agonism

**FIGURE 8:**
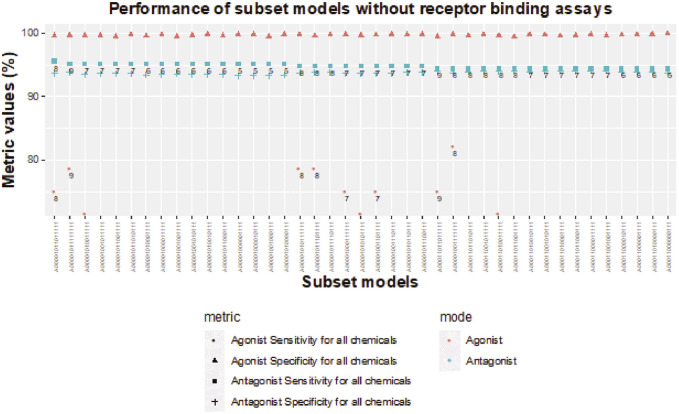
Sensitivity and specificity performance of subset models without receptor-binding assays for detecting chemicals with AR antagonism and agonism.

As noted previously, removal of all NVS receptor-binding assays does not dramatically influence sensitivity of subset models for AR antagonism, but the lack of any receptor-binding assay limits the sensitivity to 89.3% for AR agonism (Table 3). When testing chemicals for AR antagonism, there are four 5-assay subset models (A00001000001111, A00001000010111, A00001001000111, and A00001010000111) with antagonist sensitivity above 95% and antagonist specificity above 93% (Figure 8). The agonist specificity of these four 5-assay subset models is >99.5%; however, their agonist sensitivity ranges between 39% and 50%. Therefore, these subset models are suitable for the testing of AR antagonism, but they do not meet our minimum criteria for defining AR agonism.

Without receptor-binding assays, the best agonist sensitivity is available for an 8-assay subset model (A00001001111111) with antagonist sensitivity >94.4% and agonist sensitivity >82% (Figure 8). The 8-assay model contains all assays present in the first three 5-assay subset models except A7 (ATG_AR_TRANS_up), which is present in only one subset model. Interestingly, four subset models (A00001011111111, A00001010111111, A00001011101111, A00001001111111) with agonist sensitivity above 78% contain OT_AR_ARSRC1_0960, TOX21_AR_BLA_Agonist_ratio, and ACEA_AR_agonist_80h, which cover three out of the four steps in the agonist pathway outside of receptor binding. The other three assays that are always present are all antagonist assays (UPITT_HCI_U2OS_AR_TIF2_Nucleoli_Antagonist, TOX21_AR_BLA_Antagonist_ratio, and TOX21_AR_LUC_MDAKB2_Antagonist_0.5 nM_R1881), which further supports the need for binding assays to fully capture AR agonism.

### 3.7 Subset models and detection of the reference chemicals’ AR activity

We evaluated sensitivity and specificity for all subset models from Table 4 using three different sets of reference chemicals (Table 7). The first reference set is from Kleinstreuer et al. (2017). It contains 54 *in vitro* reference chemicals; however, only 44 are present in the set of 1,820 from Judson et al. (2020). The 13 antagonist subset models had specificities between 78.3% and 87%, with >95% sensitivity for antagonism detection, whereas the agonist model (A00101011101000) showed 100% sensitivity and >97% specificity for agonism detection.

**TABLE 7:** Performance of 13 antagonist subset models and one agonist subset model (A00101011101000) from the 9-assay battery on three sets of reference chemicals.

	Percentage (%)		Agonist/Antagonist	*In vitro*/*In vivo*	References
	Sensitivity	Specificity			
min	95.2	78.3	Antagonist	*In vitro*	Kleinstreuer et al. (2017)
max	95.2	87.0	Antagonist	*In vitro*	Kleinstreuer et al. (2017)
	100.0	97.2	Agonist	*In vitro*	Kleinstreuer et al. (2017)
min	95.2	79.2	Antagonist	*In vitro*	Judson et al. (2020)
max	95.2	87.5	Antagonist	*In vitro*	Judson et al. (2020)
	100.0	97.2	Agonist	*In vitro*	Judson et al. (2020)
	100.0	85.2	Antagonist	*In vivo*	Judson et al. (2020)
	100.0	100.0	Agonist	*In vivo*	Judson et al. (2020)

The second set of reference chemicals are *in vitro* reference chemicals (45 out of 46 total) from Judson et al. (2020). The agonist model had the same performance as in the case of the Kleinstreuer et al. (2017) reference chemicals, but the 13 subset models had a slightly higher prediction for antagonist specificity (79.2%–87.5%). Finally, we used the *in vivo* reference chemicals (39) from Judson et al. (2020) to test antagonism detection in 13 subset models and agonism detection in A00101011101000. Antagonism sensitivity and specificity of the *in vivo* reference chemicals were 100% and 85.2%, respectively. Both agonism sensitivity and specificity were 100%. Predictions of each subset model across each set of reference chemicals are provided in the (Supplementary Table S6).

## 4 Conclusion

We have found that the original 11-assay model (Kleinstreuer et al., 2017) and the 14-assay model (Judson et al., 2020) agree on the prediction of activity and inactivity for 1,733 out of 1,817 (>95%) chemicals in antagonist mode and 1,798 out of 1,817 (>98.9%) chemicals in agonist mode. The high level of agreement for agonist predictions is driven primarily by the inactive calls, whereas the models disagree regarding 19 chemicals and agree regarding 21 chemicals when considering only active predictions in at least one of the models. It is difficult to interpret the impact of the discrepancies because the AUC value from the *in vitro* models in these cases is lower than the AUC for any of the agonist reference chemicals. Hence, it is impossible to determine whether the differences are primarily due to false-positive or false-negative predictions. Further testing of these chemicals would increase the confidence in agonist predictions for weaker agonists and allow a better determination of the relative performance of the two models. In the short term, the agreement between the models for chemicals with higher AUC values is high, which suggests that chemicals most likely to be prioritized for Tier 1 screening would be identified using both models.

We confirmed that the agonist- and antagonist-specific assays were important for predicting agonism and antagonism, respectively. In fact, our data-driven assay selection process perfectly matched the theoretical expectations, with agonist-specific assays being highly prevalent in subset models with high agonist sensitivity and antagonist-specific assays required for high antagonist sensitivity (Figure 6). Assays corresponding to upstream processes shared between agonists and antagonists were equally prevalent in both types of models, which again met expectations. We also demonstrated that models containing assays covering the different biological events generally performed better.

We identified an optimal battery of nine assays for chemical prioritization that contains subset models for antagonism and agonism that achieve sensitivity >95% and specificity >85%. Five assays are sufficient for predicting AR antagonism and six assays for AR agonism prediction, with two assays in common. To further optimize the number of chemical-assay pairs during testing, we proposed two scenarios that tailor the number of assays used based on the *a priori* likelihood that a chemical could be an AR agonist; one is based on cheminformatics predictions of AR agonism, and the other utilizes two-stage testing. Both approaches utilize the EDSP chemical clustering performed by Nelms et al. (2023). This method of screening may be applicable to other minimal assay models as well.

Future studies will necessarily include different assays from those used for these analyses because some of the original assays are no longer available and newer methods are continually being developed (Judson et al., 2020; U.S. EPA, 2023). Based on our findings, *in vitro* batteries that substitute assays that interrogate the same biological endpoint should behave similarly to the simulations performed herein. For additional confidence, the new assays could be tested using the original ToxCast chemicals and results compared with the corresponding assays with regard to sensitivity, specificity, and variability. As new data are collected, this workflow could be used to optimize testing strategies using the currently available assays. Furthermore, as additional activity data are collected and more reference chemicals are identified, the *in silico* tools should become more reliable and eliminate the need for *in vitro* testing at the prioritization stage.

These analyses demonstrate the importance of using chemical clustering to aid the efficiency of screening approaches and demonstrate adequate coverage of the EDSP UoC. The methods described here provide an approach for finding an optimal subset assay battery from a larger set of assays in order to minimize the number of assays needed when screening for both AR antagonism and agonism. What was done in this approach to optimize screening efficiency of the AR pathway model may also be used for other molecular targets that begin with a comprehensive evaluation of many endpoints in order to determine which endpoints or assays are critical for optimal performance. This case study demonstrated the importance of evaluating both agonists and antagonists in a workflow that allowed optimal efficiency in screening. These results also show how information regarding chemical structure can be combined with preliminary data from high-throughput screening to help select chemicals for further screening.

## Data Availability

The original contributions presented in the study are included in the article/Supplementary Material, further inquiries can be directed to the corresponding author.

## Footnotes

1 The file is available at https://www.epa.gov/sites/default/files/2015-07/documents/edsp_chemical_universe_list_11_12.pdf.

## Supplementary material

The Supplementary Material for this article can be found online at: https://www.frontiersin.org/articles/10.3389/ftox.2024.1347364/full#supplementary-material. Software code used in this study can be found at: https://github.com/USEPA/edsp-ar-subset-model-analysis.
