# Supplementary material for: Optimizing androgen receptor prioritization using high-throughput assay-based activity models
Source: Front Toxicol. 2024 Mar 11;6:1347364. doi: 10.3389/ftox.2024.1347364 (PMC10961702; doi:10.3389/ftox.2024.1347364)
Supplement: Supplementary file 3 [file Table3.docx]

Supplementary Material

Optimizing androgen receptor prioritization using high-throughput assay-based activity models

Ronnie Joe Bever^1^*, Stephen W. Edwards^2^†, Todor Antonijevic^3^†, Mark D. Nelms^2^, Caroline Ring^4^, Danni Harris^2^, Scott G. Lynn^1^, David Williams^2^, Grace Chappell^5^, Rebecca Boyles^2^, Susan Borghoff^6^, Kristan J. Markey^1^

*** Correspondence:**

Ronnie Joe Bever
[Bever.Ronnie@epa.gov](mailto:Bever.Ronnie@epa.gov)

# Supplementary Tables

**Supplementary Table S1**. Table containing chemical and predicted chemical cluster information for the AR model chemicals, alongside the results from the 14-assay AR model and CoMPARA QSAR predictions. This data were used to identify clusters with potential AR activity.

Supplementary Table S2. Results from analysis comparing the 14-assay AR model from Judson et al. (2020) with the original 11-assay model from Kleinstreuer et al. (2017).

Supplemental Table S3. Hit-call matrix for 74 chemicals for which the 14-assay AR model predicts antagonist activity, but the 11-assay AR model predicts inactivity. The first two rows indicate the presence (1) or absence (0) of assays in corresponding antagonist pathways. Assays that are common between models are painted in green colons. Assays in orange columns are present only in the AR 14-assay model. Active compounds are denoted with hit-calls of 1 (blue cells) in corresponding assays, whereas inactive compounds are denoted with hit-calls of 0 (red cells).

**Supplemental Table S4**. Hit-call matrix for 7 chemicals for which the 14-assay AR model predicts agonist activity, but the 11-assay AR model predicts inactivity. The first two rows indicate the presence (1) or absence (0) of assays in corresponding antagonist pathways. Assays that are common between models are painted in green color. Assays in orange columns are present only in the AR 14-assay model. Active compounds are denoted with hit-calls of 1 (blue cells0 in corresponding assays, whereas inactive compounds are denoted with hit-call of 0 (red cells).

**Supplemental Table S5**. Results from 58,533 pair of subset models where one model predicts AR agonism and the other model predicts AR antagonism. This table provides the Hamming distance (i.e., the difference in assay content between the two models), the total number of assays required across both models, and the assay numbers.

**Supplemental Table S6**. Table containing the predictions of each agonist or antagonist subset model across each set of reference chemicals.

Supplemental Table S3. Hit-call matrix for 74 chemicals for which the 14-assay AR model predicts antagonist activity, but the 11-assay AR model predicts inactivity. The first two rows indicate the presence (1) or absence (0) of assays in corresponding antagonist pathways. Assays that are common between models are painted in green colons. Assays in orange columns are present only in the AR 14-assay model. Active compounds are denoted with hit-calls of 1 (blue cells) in corresponding assays, whereas inactive compounds are denoted with hit-calls of 0 (red cells).

| Antagonist pathway in 14-assay AR model | 1 | 1 | 1 | 1 | 1 | 1 | 0 | 0 | 0 | 0 | 0 | 1 | 1 | 1 |  |  |
| --- | --- | --- | --- | --- | --- | --- | --- | --- | --- | --- | --- | --- | --- | --- | --- | --- |
| Antagonist pathway in 11-assay AR model | 1 | 1 | 1 | 1 | 1 | 0 | 0 | 0 | 0 | 0 | 0 | 0 | 1 | 1 |  |  |
| DTXSID\ASSAY ID | A1 | A2 | A3 | A4 | A5 | A6 | A7 | A8 | A9 | A10 | A11 | A12 | A13 | A14 | Cluster ID | Hit-call in A6 or A12 |
| [DTXSID7020764](http://comptox.epa.gov/dashboard/chemical/details/DTXSID7020764) |  |  |  |  |  |  |  |  |  |  |  |  |  |  | 100 | 1 |
| [DTXSID1034187](http://comptox.epa.gov/dashboard/chemical/details/DTXSID1034187) |  |  |  |  |  |  |  |  |  |  |  |  |  |  | 419 | 1 |
| [DTXSID9021138](http://comptox.epa.gov/dashboard/chemical/details/DTXSID9021138) |  |  |  |  |  |  |  |  |  |  |  |  |  |  | 117 | 1 |
| [DTXSID4022523](http://comptox.epa.gov/dashboard/chemical/details/DTXSID4022523) |  |  |  |  |  |  |  |  |  |  |  |  |  |  | 487 | 1 |
| [DTXSID7020843](http://comptox.epa.gov/dashboard/chemical/details/DTXSID7020843) |  |  |  |  |  |  |  |  |  |  |  |  |  |  | 77 | 1 |
| [DTXSID6032562](http://comptox.epa.gov/dashboard/chemical/details/DTXSID6032562) |  |  |  |  |  |  |  |  |  |  |  |  |  |  | 158 | 1 |
| [DTXSID1020560](http://comptox.epa.gov/dashboard/chemical/details/DTXSID1020560) |  |  |  |  |  |  |  |  |  |  |  |  |  |  | 150 | 1 |
| [DTXSID9026974](http://comptox.epa.gov/dashboard/chemical/details/DTXSID9026974) |  |  |  |  |  |  |  |  |  |  |  |  |  |  | 474 | 1 |
| [DTXSID0047957](http://comptox.epa.gov/dashboard/chemical/details/DTXSID0047957) |  |  |  |  |  |  |  |  |  |  |  |  |  |  | 487 | 1 |
| [DTXSID2034881](http://comptox.epa.gov/dashboard/chemical/details/DTXSID2034881) |  |  |  |  |  |  |  |  |  |  |  |  |  |  | 214 | 1 |
| [DTXSID0041141](http://comptox.epa.gov/dashboard/chemical/details/DTXSID0041141) |  |  |  |  |  |  |  |  |  |  |  |  |  |  | 103 | 1 |
| [DTXSID4022369](http://comptox.epa.gov/dashboard/chemical/details/DTXSID4022369) |  |  |  |  |  |  |  |  |  |  |  |  |  |  | 789 | 1 |
| [DTXSID2032398](http://comptox.epa.gov/dashboard/chemical/details/DTXSID2032398) |  |  |  |  |  |  |  |  |  |  |  |  |  |  | 499 | 1 |
| [DTXSID5021332](http://comptox.epa.gov/dashboard/chemical/details/DTXSID5021332) |  |  |  |  |  |  |  |  |  |  |  |  |  |  | 602 | 1 |
| [DTXSID2026076](http://comptox.epa.gov/dashboard/chemical/details/DTXSID2026076) |  |  |  |  |  |  |  |  |  |  |  |  |  |  | NA | 1 |
| [DTXSID0022777](http://comptox.epa.gov/dashboard/chemical/details/DTXSID0022777) |  |  |  |  |  |  |  |  |  |  |  |  |  |  | 296 | 1 |
| [DTXSID8027793](http://comptox.epa.gov/dashboard/chemical/details/DTXSID8027793) |  |  |  |  |  |  |  |  |  |  |  |  |  |  | 287 | 1 |
| [DTXSID0021460](http://comptox.epa.gov/dashboard/chemical/details/DTXSID0021460) |  |  |  |  |  |  |  |  |  |  |  |  |  |  | 565 | 1 |
| [DTXSID9022312](http://comptox.epa.gov/dashboard/chemical/details/DTXSID9022312) |  |  |  |  |  |  |  |  |  |  |  |  |  |  | 179 | 1 |
| [DTXSID4048195](http://comptox.epa.gov/dashboard/chemical/details/DTXSID4048195) |  |  |  |  |  |  |  |  |  |  |  |  |  |  | 200 | 1 |
| [DTXSID4024274](http://comptox.epa.gov/dashboard/chemical/details/DTXSID4024274) |  |  |  |  |  |  |  |  |  |  |  |  |  |  | 103 | 1 |
| [DTXSID6020220](http://comptox.epa.gov/dashboard/chemical/details/DTXSID6020220) |  |  |  |  |  |  |  |  |  |  |  |  |  |  | 475 | 1 |
| [DTXSID2037714](http://comptox.epa.gov/dashboard/chemical/details/DTXSID2037714) |  |  |  |  |  |  |  |  |  |  |  |  |  |  | 179 | 1 |
| [DTXSID6047288](http://comptox.epa.gov/dashboard/chemical/details/DTXSID6047288) |  |  |  |  |  |  |  |  |  |  |  |  |  |  | 457 | 1 |
| [DTXSID3024104](http://comptox.epa.gov/dashboard/chemical/details/DTXSID3024104) |  |  |  |  |  |  |  |  |  |  |  |  |  |  | 691 | 1 |
| [DTXSID6032647](http://comptox.epa.gov/dashboard/chemical/details/DTXSID6032647) |  |  |  |  |  |  |  |  |  |  |  |  |  |  | 145 | 1 |
| [DTXSID1032648](http://comptox.epa.gov/dashboard/chemical/details/DTXSID1032648) |  |  |  |  |  |  |  |  |  |  |  |  |  |  | 715 | 1 |
| [DTXSID9020114](http://comptox.epa.gov/dashboard/chemical/details/DTXSID9020114) |  |  |  |  |  |  |  |  |  |  |  |  |  |  | 113 | 1 |
| [DTXSID4022446](http://comptox.epa.gov/dashboard/chemical/details/DTXSID4022446) |  |  |  |  |  |  |  |  |  |  |  |  |  |  | 152 | 1 |
| [DTXSID1040611](http://comptox.epa.gov/dashboard/chemical/details/DTXSID1040611) |  |  |  |  |  |  |  |  |  |  |  |  |  |  | NA | 1 |
| [DTXSID2037712](http://comptox.epa.gov/dashboard/chemical/details/DTXSID2037712) |  |  |  |  |  |  |  |  |  |  |  |  |  |  | 439 | 1 |
| [DTXSID7042273](http://comptox.epa.gov/dashboard/chemical/details/DTXSID7042273) |  |  |  |  |  |  |  |  |  |  |  |  |  |  | 474 | 1 |
| [DTXSID0041357](http://comptox.epa.gov/dashboard/chemical/details/DTXSID0041357) |  |  |  |  |  |  |  |  |  |  |  |  |  |  | 82 | 1 |
| [DTXSID1047368](http://comptox.epa.gov/dashboard/chemical/details/DTXSID1047368) |  |  |  |  |  |  |  |  |  |  |  |  |  |  | 785 | 1 |
| [DTXSID1047283](http://comptox.epa.gov/dashboard/chemical/details/DTXSID1047283) |  |  |  |  |  |  |  |  |  |  |  |  |  |  | 785 | 1 |
| [DTXSID3042390](http://comptox.epa.gov/dashboard/chemical/details/DTXSID3042390) |  |  |  |  |  |  |  |  |  |  |  |  |  |  | 49 | 1 |
| [DTXSID7024245](http://comptox.epa.gov/dashboard/chemical/details/DTXSID7024245) |  |  |  |  |  |  |  |  |  |  |  |  |  |  | 130 | 1 |
| [DTXSID0048185](http://comptox.epa.gov/dashboard/chemical/details/DTXSID0048185) |  |  |  |  |  |  |  |  |  |  |  |  |  |  | 165 | 1 |
| [DTXSID7047358](http://comptox.epa.gov/dashboard/chemical/details/DTXSID7047358) |  |  |  |  |  |  |  |  |  |  |  |  |  |  | 396 | 1 |
| [DTXSID5020233](http://comptox.epa.gov/dashboard/chemical/details/DTXSID5020233) |  |  |  |  |  |  |  |  |  |  |  |  |  |  | 298 | 1 |
| [DTXSID4020375](http://comptox.epa.gov/dashboard/chemical/details/DTXSID4020375) |  |  |  |  |  |  |  |  |  |  |  |  |  |  | 752 | 1 |
| [DTXSID7040362](http://comptox.epa.gov/dashboard/chemical/details/DTXSID7040362) |  |  |  |  |  |  |  |  |  |  |  |  |  |  | 168 | 1 |
| [DTXSID0035748](http://comptox.epa.gov/dashboard/chemical/details/DTXSID0035748) |  |  |  |  |  |  |  |  |  |  |  |  |  |  | 651 | 1 |
| [DTXSID2045232](http://comptox.epa.gov/dashboard/chemical/details/DTXSID2045232) |  |  |  |  |  |  |  |  |  |  |  |  |  |  | 720 | 1 |
| [DTXSID4022525](http://comptox.epa.gov/dashboard/chemical/details/DTXSID4022525) |  |  |  |  |  |  |  |  |  |  |  |  |  |  | 423 | 1 |
| [DTXSID4024066](http://comptox.epa.gov/dashboard/chemical/details/DTXSID4024066) |  |  |  |  |  |  |  |  |  |  |  |  |  |  | 15 | 1 |
| [DTXSID9020372](http://comptox.epa.gov/dashboard/chemical/details/DTXSID9020372) |  |  |  |  |  |  |  |  |  |  |  |  |  |  | 752 | 1 |
| [DTXSID9020293](http://comptox.epa.gov/dashboard/chemical/details/DTXSID9020293) |  |  |  |  |  |  |  |  |  |  |  |  |  |  | 463 | 1 |
| [DTXSID8021301](http://comptox.epa.gov/dashboard/chemical/details/DTXSID8021301) |  |  |  |  |  |  |  |  |  |  |  |  |  |  | 419 | 1 |
| [DTXSID5023950](http://comptox.epa.gov/dashboard/chemical/details/DTXSID5023950) |  |  |  |  |  |  |  |  |  |  |  |  |  |  | 566 | 1 |
| [DTXSID7020267](http://comptox.epa.gov/dashboard/chemical/details/DTXSID7020267) |  |  |  |  |  |  |  |  |  |  |  |  |  |  | 150 | 1 |
| [DTXSID8022377](http://comptox.epa.gov/dashboard/chemical/details/DTXSID8022377) |  |  |  |  |  |  |  |  |  |  |  |  |  |  | 789 | 1 |
| [DTXSID1021087](http://comptox.epa.gov/dashboard/chemical/details/DTXSID1021087) |  |  |  |  |  |  |  |  |  |  |  |  |  |  | 732 | 1 |
| [DTXSID1040794](http://comptox.epa.gov/dashboard/chemical/details/DTXSID1040794) |  |  |  |  |  |  |  |  |  |  |  |  |  |  | 570 | 1 |
| [DTXSID1048122](http://comptox.epa.gov/dashboard/chemical/details/DTXSID1048122) |  |  |  |  |  |  |  |  |  |  |  |  |  |  | NA | 1 |
| [DTXSID5032315](http://comptox.epa.gov/dashboard/chemical/details/DTXSID5032315) |  |  |  |  |  |  |  |  |  |  |  |  |  |  | 372 | 1 |
| [DTXSID8021224](http://comptox.epa.gov/dashboard/chemical/details/DTXSID8021224) |  |  |  |  |  |  |  |  |  |  |  |  |  |  | 568 | 0 |
| [DTXSID1034634](http://comptox.epa.gov/dashboard/chemical/details/DTXSID1034634) |  |  |  |  |  |  |  |  |  |  |  |  |  |  | 214 | 1 |
| [DTXSID5035589](http://comptox.epa.gov/dashboard/chemical/details/DTXSID5035589) |  |  |  |  |  |  |  |  |  |  |  |  |  |  | 246 | 1 |
| [DTXSID7032393](http://comptox.epa.gov/dashboard/chemical/details/DTXSID7032393) |  |  |  |  |  |  |  |  |  |  |  |  |  |  | 563 | 1 |
| [DTXSID0047371](http://comptox.epa.gov/dashboard/chemical/details/DTXSID0047371) |  |  |  |  |  |  |  |  |  |  |  |  |  |  | 448 | 1 |
| [DTXSID4020373](http://comptox.epa.gov/dashboard/chemical/details/DTXSID4020373) |  |  |  |  |  |  |  |  |  |  |  |  |  |  | 752 | 1 |
| [DTXSID9032329](http://comptox.epa.gov/dashboard/chemical/details/DTXSID9032329) |  |  |  |  |  |  |  |  |  |  |  |  |  |  | 795 | 1 |
| [DTXSID5020811](http://comptox.epa.gov/dashboard/chemical/details/DTXSID5020811) |  |  |  |  |  |  |  |  |  |  |  |  |  |  | 138 | 1 |
| [DTXSID2020688](http://comptox.epa.gov/dashboard/chemical/details/DTXSID2020688) |  |  |  |  |  |  |  |  |  |  |  |  |  |  | 734 | 1 |
| [DTXSID3021770](http://comptox.epa.gov/dashboard/chemical/details/DTXSID3021770) |  |  |  |  |  |  |  |  |  |  |  |  |  |  | 411 | 1 |
| [DTXSID8021771](http://comptox.epa.gov/dashboard/chemical/details/DTXSID8021771) |  |  |  |  |  |  |  |  |  |  |  |  |  |  | 474 | 1 |
| [DTXSID5020867](http://comptox.epa.gov/dashboard/chemical/details/DTXSID5020867) |  |  |  |  |  |  |  |  |  |  |  |  |  |  | 95 | 1 |
| [DTXSID6041422](http://comptox.epa.gov/dashboard/chemical/details/DTXSID6041422) |  |  |  |  |  |  |  |  |  |  |  |  |  |  | 408 | 1 |
| [DTXSID2036112](http://comptox.epa.gov/dashboard/chemical/details/DTXSID2036112) |  |  |  |  |  |  |  |  |  |  |  |  |  |  | 101 | 1 |
| [DTXSID2026602](http://comptox.epa.gov/dashboard/chemical/details/DTXSID2026602) |  |  |  |  |  |  |  |  |  |  |  |  |  |  | 475 | 1 |
| [DTXSID2020426](http://comptox.epa.gov/dashboard/chemical/details/DTXSID2020426) |  |  |  |  |  |  |  |  |  |  |  |  |  |  | 241 | 1 |
| [DTXSID5047328](http://comptox.epa.gov/dashboard/chemical/details/DTXSID5047328) |  |  |  |  |  |  |  |  |  |  |  |  |  |  | 432 | 1 |
| [DTXSID1047366](http://comptox.epa.gov/dashboard/chemical/details/DTXSID1047366) |  |  |  |  |  |  |  |  |  |  |  |  |  |  | 576 | 1 |
