# Supplementary material for: Optimizing androgen receptor prioritization using high-throughput assay-based activity models
Source: Front Toxicol. 2024 Mar 11;6:1347364. doi: 10.3389/ftox.2024.1347364 (PMC10961702; doi:10.3389/ftox.2024.1347364)
Supplement: Supplementary file 5 [file Table4.docx]

Supplementary Material

Optimizing androgen receptor prioritization using high-throughput assay-based activity models

Ronnie Joe Bever^1^*, Stephen W. Edwards^2^†, Todor Antonijevic^3^†, Mark D. Nelms^2^, Caroline Ring^4^, Danni Harris^2^, Scott G. Lynn^1^, David Williams^2^, Grace Chappell^5^, Rebecca Boyles^2^, Susan Borghoff^6^, Kristan J. Markey^1^

*** Correspondence:**

Ronnie Joe Bever
[Bever.Ronnie@epa.gov](mailto:Bever.Ronnie@epa.gov)

Supplementary Table S4. Hit-call matrix for 7 chemicals for which the 14-assay AR model predicts agonist activity, but the 11-assay AR model predicts inactivity. The first two rows indicate the presence (1) or absence (0) of assays in corresponding antagonist pathways. Assays that are common between models are painted in green color. Assays in orange columns are present only in the AR 14-assay model. Active compounds are denoted with hit-calls of 1 (blue cells0 in corresponding assays, whereas inactive compounds are denoted with hit-call of 0 (red cells).

| Agonist pathway in 14-assay AR model | 1 | 1 | 1 | 1 | 1 | 1 | 1 | 1 | 1 | 1 | 1 | 0 | 0 | 0 |  | |  | |
| --- | --- | --- | --- | --- | --- | --- | --- | --- | --- | --- | --- | --- | --- | --- | --- | --- | --- | --- |
| Agonist pathway in 11-assay AR model | 1 | 1 | 1 | 1 | 1 | 0 | 1 | 1 | 1 | 1 | 0 | 0 | 0 | 0 |  | |  | |
| DTXSID\ASSAY ID | A1 | A2 | A3 | A4 | A5 | A6 | A7 | A8 | A9 | A10 | A11 | A12 | A13 | A14 | Cluster ID | Hit-calls in A6 or A11 | |  |
| [DTXSID8047347](http://comptox.epa.gov/dashboard/chemical/details/DTXSID8047347) |  |  |  |  |  |  |  |  |  |  |  |  |  |  | 188 | 1 | |  |
| [DTXSID5020576](http://comptox.epa.gov/dashboard/chemical/details/DTXSID5020576) |  |  |  |  |  |  |  |  |  |  |  |  |  |  | 789 | 1 | |  |
| [DTXSID5024845](http://comptox.epa.gov/dashboard/chemical/details/DTXSID5024845) |  |  |  |  |  |  |  |  |  |  |  |  |  |  | 694 | 1 | |  |
| [DTXSID7044718](http://comptox.epa.gov/dashboard/chemical/details/DTXSID7044718) |  |  |  |  |  |  |  |  |  |  |  |  |  |  | 493 | 1 | |  |
| [DTXSID2020894](http://comptox.epa.gov/dashboard/chemical/details/DTXSID2020894) |  |  |  |  |  |  |  |  |  |  |  |  |  |  | 113 | 1 | |  |
| [DTXSID4020664](http://comptox.epa.gov/dashboard/chemical/details/DTXSID4020664) |  |  |  |  |  |  |  |  |  |  |  |  |  |  | 153 | 1 | |  |
| [DTXSID4047333](http://comptox.epa.gov/dashboard/chemical/details/DTXSID4047333) |  |  |  |  |  |  |  |  |  |  |  |  |  |  | 198 | 0 | |  |
